# The longitudinal interplay between acculturation and intergroup contact in adolescence

**DOI:** 10.1111/bjso.70129

**Published:** 2026-09-02

**Authors:** Lucia Jochym Illy, Beatrice Bobba, Francesca Prati, Monica Rubini, Elisabetta Crocetti

**Affiliations:** ^1^ Department of Psychology University of Bologna Bologna Italy; ^2^ Department of Interdisciplinary Social Science Utrecht University Utrecht the Netherlands

**Keywords:** acculturation, adolescents, intergroup contact, random‐intercept cross‐lagged modelling

## Abstract

In current multicultural societies, mutual adjustment of majority and ethnic minority groups is a crucial issue shaped by groups' acculturation preferences and their encounters. These aspects are particularly relevant in adolescence when young people extend their social relationships and develop attitudes towards others. However, the association between acculturation preferences across domains and the quantity and quality of intergroup contact in adolescence remains underexplored. Thus, we conducted a three‐wave longitudinal study with majority (*N* = 740; 64.73% female; *M*_age_ = 14.58) and ethnic minority (*N* = 244; 56.56% female; *M*_age_ = 14.91) adolescents to examine these links. Results of random‐intercept cross‐lagged models showed that among majority adolescents, at the between‐person level, their preferences for minority acculturation were mainly associated with contact quality in and out‐of‐school, with preference for heritage culture maintenance linked to more positive contact and preference for adoption linked to more negative contact. Within‐person increases in positive contact in school were followed by decreases in adoption preference. Among ethnic minority adolescents, at the between‐person level, their adoption preference was related to more frequent and positive contact across contexts, with additional within‐person associations. Overall, the findings emphasize the context‐specific and asymmetrical interplay between acculturation and intergroup contact in adolescence.

Modern societies have grown increasingly diverse due to rising migration over recent decades (Boin et al., 2021). Immigration in Italy has increased significantly in the 21st century, reaching an immigrant population of 5.3 million by 2025 (Istituto Nazionale di Statistica, 2025). This increasing demographic diversity is also reflected in the adolescent population. Since the early 2000s, the share of non‐Italian students has nearly doubled, and as of the 2022/2023 school year, nearly one in twelve high school students in Italy holds a non‐Italian citizenship (Ministero dell'Istruzione e del Merito, 2024). In such multicultural contexts, adolescents navigate intergroup interactions in everyday settings, where cultural practices, values and identities are negotiated and become salient (Crocetti et al., 2024). These contexts involve challenges for both majority (receiving society), and ethnic minority adolescents, as they adjust to one another within shared social environments, such as schools and leisure contexts (Berry, 2005). Accordingly, intergroup dynamics, such as intergroup contact seeking and avoidance may depend on majority and ethnic minority adolescents' preferences regarding how ingroup and outgroup cultural adjustment should be approached in the host society (i.e. acculturation preferences; Berry, 2005; Piontkowski et al., 2000). Nevertheless, acculturation preferences are shaped by power asymmetries (Bierwiaczonek & Kunst, 2021), such that the majority group often exerts greater influence on minority groups' acculturation than vice versa; thus, majority preferences for minority acculturation remain a key aspect in understanding majority–minority dynamics (Bourhis et al., 1997). Moreover, adolescence is a particularly critical period for examining these processes, as social identities, intergroup attitudes and patterns of peer interaction are still forming and may influence one another over time (Crocetti et al., 2023; Rutland et al., 2010).

Therefore, to address the current issue of majority–minority cultural adjustment and intergroup dynamics in increasingly diverse societies, it is crucial to understand the role of acculturation preferences on contact seeking and avoidance between adolescents from both groups' perspectives. In this regard, research on acculturation and intergroup contact has largely developed in parallel, with the former focusing primarily on its effects on well‐being of ethnic minorities (e.g. Schwarz & Pfammatter, 2024; Uskul et al., 2024) and the latter on the role of intergroup contact in reducing prejudice among majority groups (e.g. Binder et al., 2009; Van Assche et al., 2023). Therefore, much empirical work has treated intergroup contact as an antecedent of acculturation preferences and related outcomes (e.g. Bagci et al., 2018; Van Acker & Vanbeselaere, 2011; Zagefka et al., 2014). However, acculturation preferences indicate personal orientations towards cultural diversity, ranging from desires of reciprocal openness and engagement with other cultures members to tendencies towards distancing or avoidance, which may reflect contact seeking and avoidance, resulting in the quantity and quality of one's intergroup contact (Berry, 2005; Bourhis et al., 1997). Consistent with this perspective, studies on acculturation with adult samples suggest that majority group members' acculturation preferences are linked with intergroup attitudes and behavioural tendencies towards ethnic minority groups, including social distance intentions and discriminatory behaviour (e.g. Geschke et al., 2010; Zick et al., 2001). While these lines of research have offered valuable insights, they leave unanswered questions, regarding whether and which acculturation preferences of both majority and ethnic minority groups' adolescents facilitate or hinder intergroup encounters over time. Although few recent longitudinal studies have examined temporal associations between acculturation and intergroup contact among adolescents, the consistency (or differences) of these patterns across multiple everyday contexts (i.e. in school and out‐of‐school) for both majority and ethnic minority groups remains less clear. The present study seeks to extend this work by assessing the longitudinal interplay between acculturation preferences (i.e. heritage culture maintenance, destination culture adoption) and contact seeking and avoidance (i.e. the quantity and quality of intergroup contact) across school and out‐of‐school contexts. These settings differ substantially in their degree of institutional structure: school contact is structurally constrained and regulated, involving repeated exposure to diverse peers, whereas out‐of‐school contact is more voluntary and personally chosen, making contact patterns more reflective of individual acculturation preferences and friendship‐selection processes (Dixon et al., 2005; Thijs & Verkuyten, 2014). Acculturation preferences may therefore shape intergroup contact patterns differently across these two contexts. The present study addresses these paths among both majority and ethnic minority adolescents in Italy, foregrounding both groups' preferences for ethnic minority members acculturation as antecedents of subsequent intergroup contact patterns.

## Acculturation of majority and ethnic minority groups

The process of acculturation involves changes at the individual and group level in terms of cultural practices, values and identity that occur through ongoing exchanges with another culture (Sam & Berry, 2006). Over the years, there have been multiple models of acculturation (Berry, 1974; Bourhis et al., 1997; Gordon, 1964; Navas et al., 2005). A simplistic unidimensional view of integration as a one‐way assimilation process (Gordon, 1964) has been progressively replaced by a bidimensional approach. Berry's bidimensional model (1974) conceptualized how ethnic minorities navigate acculturation by considering two independent dimensions: heritage culture maintenance and destination culture adoption. The intersection of these dimensions defines four acculturation strategies: assimilation (i.e. relinquishing heritage culture in favour of the destination culture), integration (i.e. maintaining elements of heritage culture while adopting elements of the destination culture), separation (i.e. maintaining heritage culture and minimizing contact with the dominant culture) and marginalization (i.e. low identification with both the heritage and destination culture). Several studies have shown that integration is associated with higher well‐being among ethnic minority groups (e.g. Berry, 1997; Schwartz et al., 2010).

Berry's bidimensional model was developed into more complex models (i.e. Interactive Acculturation Model, Bourhis et al., 1997; Relative Acculturation Extended Model, Navas et al., 2005). In this regard, the Relative Acculturation Extended Model suggests that acculturation concerns both the majority and ethnic minority groups' acculturation preferences, perceptions and expectations, which vary across life domains (i.e. central and peripheral). Central domains refer to private contexts of life (i.e. family, religious beliefs and traditions, principles and values), while peripheral domains concern the broader public and social contexts (i.e. school, consumption habits and friendships; Navas et al., 2005).

Building on this model, acculturation research has examined how majority and ethnic minority groups differ and overlap in their acculturation preferences across domains. This body of work shows that preferences regarding heritage culture maintenance and destination culture adoption are neither uniform nor fixed but instead vary depending on the group and domain under consideration (e.g. Arends‐Tóth & van de Vijver, 2006; López‐Rodríguez et al., 2014; Navas et al., 2007; Zagefka et al., 2014). Specifically, in central domains, majority group members usually prefer destination culture adoption of ethnic minority group members, whereas ethnic minority members prefer to maintain their heritage culture more (e.g. Mancini & Bottura, 2014; Navas et al., 2007). In peripheral domains, both groups typically have a stronger preference for destination culture adoption by ethnic minority groups (Navas et al., 2007). Notably, until recently in most research on acculturation, majority members' acculturation preferences usually concern expectations regarding others, whereas ethnic minority members' preferences relate to their own cultural practices and identities (Zagefka et al., 2023), even if Berry's (1974) bidimensional model already highlighted the reciprocal influence of both groups on each other's acculturation.

Taken together, theoretical frameworks highlight acculturation as a multifaceted and context‐dependent process. Nevertheless, empirical research has often examined group perspectives in isolation, mostly focusing on the role of ethnic minority groups' acculturation preferences (for a more comprehensive overview, see Zagefka et al., 2023) on specific outcomes (e.g. well‐being; e.g. Berry & Hou, 2017; Schwarz & Pfammatter, 2024; Uskul et al., 2024). Thus, less attention has been paid to how acculturation preferences for the minority group relate to intergroup contact patterns of both majority and minority group members in current multicultural societies.

## Patterns of intergroup contact

Intergroup contact refers to interactions between members of different social, ethnic or cultural groups (Dovidio et al., 2008). According to intergroup contact theory, positive contact between members of different groups can reduce outgroup prejudice and improve intergroup relations (Allport, 1954). In modern societies, intergroup contact commonly occurs in everyday social settings and varies both in quantity (i.e. frequency) and quality, encompassing positive and negative experiences (Graf et al., 2014). While positive contact typically refers to meaningful, friendly intergroup encounters (Pettigrew & Tropp, 2006), negative contact involves discriminatory, conflictual or hostile interactions (Barlow et al., 2012).

However, intergroup contact is not merely a matter of passive exposure. Rather, engagement in intergroup contact reflects how individuals seek, navigate or distance themselves from outgroup members, making it an active and dynamic process (Paolini et al., 2018). Over time, repeated patterns of intergroup contact quantity and quality emerge from these interactional tendencies and are systematically linked to individuals' willingness to seek out future intergroup contact encounters (Paolini et al., 2014).

Most intergroup contact research has primarily examined its consequences on intergroup attitudes, such as prejudice reduction and improved outgroup evaluations (for a meta‐analysis see, Pettigrew & Tropp, 2006). While positive contact is typically associated with increased openness and seeking of future intergroup interactions, along with greater acceptance of other cultures (Paolini et al., 2018), negative contact tends to increase intergroup anxiety and promote outgroup avoidance among both majority (Meleady & Forder, 2018) and ethnic minority group members (Árnadóttir et al., 2023; Prati et al., 2024). Longitudinal studies further suggest that intergroup contact and attitudes are related over time, with evidence of reciprocal associations between contact and prejudice (Binder et al., 2009). However, traditional cross‐lagged models may not adequately capture within‐person change, potentially limiting the interpretation of these findings (Hamaker et al., 2015).

While this line of research offers meaningful insights into the relation between intergroup contact and attitudes, comparatively less attention has been paid to how intergroup contact patterns are embedded within acculturation processes. Cross‐sectional studies with adult samples have tested this link in specific contexts (e.g. workplace; Vezzali & Giovannini, 2014), along with its moderators, such as perceived discrimination (te Lindert et al., 2022). These studies showed that an increase in the quantity of intergroup contact is linked to majority group members' preferences for both heritage culture maintenance and the adoption of destination culture by ethnic minority members (i.e. integration). From the perspective of ethnic minority group members, the increase in contact quantity is typically related to their preference for destination culture adoption.

In conclusion, research on intergroup contact has substantially advanced the understanding of its benefits. However, less attention has been paid to the extent to which personal tendencies such as acculturation preferences in current multicultural societies may shape subsequent patterns of intergroup contact. Addressing this question is particularly important for understanding intergroup dynamics, particularly in the crucial phase of adolescence.

## Acculturation and intergroup contact in adolescence

Adolescence is a critical period for developing social attitudes and identities (Cook & Bird, 2011; Crocetti et al., 2023; Crocetti, 2026). Developmental changes in cognitive abilities and the increased importance of social relationships make adolescents particularly receptive to social and group norms and expectations (Hjerm et al., 2018; Karataş, 2024; Merrilees et al., 2023). As a result, acculturation processes and intergroup relations remain relatively malleable during adolescence, particularly in increasingly multicultural societies, where exposure to ethnically diverse peers is becoming more common (Killen et al., 2013).

From a contextual perspective, schools constitute primary settings in which adolescents experience acculturation processes (Horenczyk & Tatar, 2012; Karataş et al., 2020) and repeated intergroup contact (Thijs & Verkuyten, 2014). In schools, majority and ethnic minority adolescents encounter one another on a regular basis, making these contexts central spaces for the negotiation of cultural identities and intergroup relations over time (Rutland et al., 2010). Through curricula, institutional practices and peer interactions, schools convey societal norms and expectations regarding cultural diversity (Karataş, Eckstein, et al., 2023b) therefore shaping acculturation‐related orientations and preferences among adolescents from both groups (Civitillo et al., 2017; Karataş, 2024). As structured social environments, schools constrain and repeatedly enact opportunities for majority–minority contact (Thijs & Verkuyten, 2014). Then, adolescents' acculturation preferences may shape the extent to which they engage in or withdraw from these contact opportunities. Within these institutional contexts, majority group adolescents have greater influence over interaction norms (Thijs & Verkuyten, 2014), developing expectations regarding ethnic minority peers' cultural adjustment, whereas ethnic minority adolescents negotiate their own cultural identities and preferences, with schools potentially supporting or constraining their heritage culture maintenance (Motti‐Stefanidi & Masten, 2017).

Beyond school, adolescents also increasingly expand their social worlds into out‐of‐school contexts, including extracurricular activities, community‐based activities and peer and social groups (Mahoney et al., 2009; Pagano et al., 2024). Participation in these contexts is typically less institutionally structured and may more strongly reflect personal social choices, opportunity structures and resource availability, such as neighbourhood composition and access to organized activities (Dixon et al., 2005). Evidence further suggests that intergroup contact experiences can spill over across contexts, such that contact patterns in school are usually associated with contact patterns out‐of‐school, particularly in terms of their quality (i.e. *valence consistent spillover effect*; Karataş, Rubini, et al., 2023). This underscores the importance of considering multiple socialization environments when examining acculturation processes and intergroup contact patterns during adolescence.

Despite growing attention to acculturation processes in adolescence, few studies have examined the link between acculturation and intergroup contact among adolescents, especially in the long‐term (Gieling et al., 2014; González et al., 2017; Hässler et al., 2018; Inguglia et al., 2020; Van Acker & Vanbeselaere, 2011). Existing work has primarily focused on how intergroup contact is related to acculturation or acculturation‐related attitudes. For instance, a cross‐sectional study among Dutch adolescents found a positive link between contact quantity and greater tolerance of minority group members' (Muslim immigrants) cultural practices (Gieling et al., 2014). Two studies conducted in the Italian context examined intercultural relations among Tunisian (ethnic minority) and Italian (majority) adolescents (Inguglia et al., 2020). Among majority adolescents, more contact with Tunisian peers was linked to greater endorsement of multiculturalism and lower support for segregation, suggesting an overall openness towards integration (both maintenance of heritage culture and adoption of the host culture). Among ethnic minority adolescents, contact with Italian peers was linked to stronger preferences for integration, which, in turn, was related to the best psychosocial outcomes. Furthermore, a study with Flemish majority adolescents showed that their perceptions of Turkish immigrants' (ethnic minority group) preferences for maintenance of their heritage culture without adopting the host culture were related to their negative feelings towards the ethnic minority group (Van Acker & Vanbeselaere, 2011). Moreover, positive contact with the minority group and perceived integration efforts was related to lower negative emotions, and higher tolerance towards the outgroup, whereas contact quantity alone showed no effect. Extending this work longitudinally, a school‐based study with indigenous and non‐indigenous students in Chile demonstrated that quality of intergroup contact and peer norms supporting cross‐group friendships were associated with changes in acculturation‐related attitudes and identity processes over time (González et al., 2017). Similarly, in another longitudinal study with adolescents, intergroup contact quantity and quality were related to changes in majority adolescents' acculturation preferences over time, with some evidence for reverse pathways (i.e. from acculturation preferences to contact), although weaker or marginal (Hässler et al., 2018).

Overall, these studies provide evidence of how intergroup contact shapes acculturation during adolescence, especially among majority groups. However, less is known about whether and how acculturation preferences themselves shape both majority and minority adolescents' intergroup contact patterns over time. Acculturation preferences as personal orientations towards cultural diversity may translate into approach and avoidance tendencies that directly shape the quantity and quality of intergroup encounters among majority group members: preferences implying acceptance of cultural diversity may facilitate contact seeking, whereas preferences reflecting expectations of cultural assimilation may promote avoidance (Bourhis et al., 1997; Piontkowski et al., 2000). Yet empirical evidence on this direction, particularly in adolescence, remains limited. Is it only contact that fosters acculturation preferences among different groups or can pre‐existing acculturation preferences shape intergroup contact experiences?

## The present study

Addressing the gaps in current literature on acculturation and intergroup contact, in the light of the scarce research on the antecedents of contact seeking and avoidance (see Kauff et al., 2021), this study aims to assess longitudinal associations between adolescents' acculturation preferences in central (e.g. family, values) and peripheral domains (e.g. school, friendships), and intergroup contact patterns, in terms of quantity and quality of intergroup contact in and out‐of‐school contexts, both at the between‐person and within‐person level. These associations are examined separately for majority and ethnic minority adolescents living in Italy. Previous research suggests that majority and minority groups can differ in their acculturation preferences, but also share some areas of overlap, particularly regarding destination culture adoption in public domains. In general, majority groups tend to prefer ethnic minority groups to adopt the host culture, whereas ethnic minority groups tend to show a higher preference for maintaining their heritage culture (Arends‐Tóth & van de Vijver, 2006; Navas et al., 2007). The present hypotheses are informed by these broader patterns. Specifically, acculturation preferences that are misaligned with preferences commonly endorsed by the other group should be associated with less favourable intergroup contact experiences. Thus, the following hypotheses, which were not pre‐registered, were formulated.

### Majority group

Focusing on the links between specific acculturation preferences and types of intergroup contact, majority group adolescents' preference for minority groups to maintain their heritage culture, reflecting a more accepting stance towards ethnic minorities' heritage culture and traditions (Gieling et al., 2014), is expected to be associated with increased positive contact (*Hypothesis 1a*) and with reduced negative contact (*Hypothesis 1b*) with ethnic minority adolescents over time. Moreover, majority group adolescents' preference for minority groups to adopt the destination culture, reflecting greater support for cultural adaptation to the receiving society (Mancini & Bottura, 2014), is expected to be associated with reduced positive contact (*Hypothesis 2a*) and with increased negative contact (*Hypothesis 2b*) with ethnic minority adolescents over time. Given that schools are structured environments with institutional norms that support, or at least allow for, inclusion and cultural pluralism, they provide facilitating conditions for promoting intergroup contact and its benefits (Allport, 1954; Karataş, Eckstein, et al., 2023b). Accordingly, acculturation preferences that are more supportive of cultural pluralism may be related to intergroup contact in school to a higher extent than in out‐of‐school contexts. Thus, majority adolescents' preference for heritage culture maintenance is hypothesized to be associated with intergroup contact quantity in school to a higher extent than out‐of‐school (*Hypothesis 3*). This pattern is expected among majority group adolescents, who typically exert greater normative influence over intergroup interactions in school, whereas ethnic minority adolescents and their preferences often have less control over contact opportunities in schools (Motti‐Stefanidi & Masten, 2017; Thijs & Verkuyten, 2014). All the hypotheses will be tested for acculturation preferences related to central and peripheral domains separately, exploring the consistency of the results across them.

The bidirectionality of the acculturation‐contact associations will be explored. Although prior longitudinal evidence has predominantly shown stronger associations from contact to acculturation preferences than the reverse (e.g. González et al., 2017; Hässler et al., 2018) this pattern has not been systematically examined separately for majority and minority groups and multiple contexts. Given that majority adolescents have greater freedom to choose whether to interact or avoid intergroup interactions (Lefringhausen et al., 2022), we exploratorily examined whether majority group members' acculturation preferences would be associated with subsequent intergroup contact quality over time to a higher extent than the reverse pattern (*Hypothesis 4*).

### Ethnic minority group

Building on research suggesting that majority group members often prefer some degree of destination culture adoption by ethnic minority groups (Arends‐Tóth & van de Vijver, 2004, 2006), ethnic minority group adolescents' preferences for heritage culture maintenance are expected to be associated with reduced positive contact (*Hypothesis 5a*) and with increased negative contact (*Hypothesis 5b*) with the majority adolescents over time. Following the same reasoning, ethnic minority members' preference for destination culture adoption is expected to be associated with increased positive contact (*Hypothesis 6a*) and reduced negative contact (*Hypothesis 6b*) with majority adolescents over time. As for the majority group, all the hypotheses will be tested for acculturation preferences related to central and peripheral domains separately, exploring the consistency of the results across them.

The bidirectionality of the acculturation‐contact associations will be explored as well. However, given that minority members often have fewer possibilities to avoid the majority group (Stathi et al., 2020), intergroup contact quantity and quality are expected to be associated with subsequent acculturation preferences of ethnic minority members to a higher extent than the reverse pattern (*Hypothesis 7*).

## METHODS

### Participants

Participants of the present study were drawn from the longitudinal research project ‘Developing Inclusive Identities in Adolescence’,^1^ which included three assessments (T1–T3) with a six‐month interval (see Karataş, Eckstein, et al., 2023a, 2023b). For the purpose of the current study, only adolescents who completed at least two assessments were included. This yielded a final longitudinal sample^2^ of 984 adolescents (62.70% female; *M*_age_ = 14.66, *SD*_age_ = 0.74), of which 740 Italian majority (64.73% female; *M*_age_ = 14.58; *SD*_age_ = 0.68) and 244 ethnic minority (56.56% female; *M*_age_ = 14.91; *SD*_age_ = 0.84) participants. Adolescents were in their first year of high school at T1 and their second year at T2 and T3.

Participants were enrolled in 48 different classrooms across 7 schools (see Karataş, Eckstein, et al., 2023a, 2023b for further details). Almost all participants were recruited from multi‐ethnic classrooms, where the average percentage of ethnic minority adolescents was 24.8%. Most participants came from two‐parent families (76.93%), the others reported being in separated or divorced families (21.65%) or in other family situations (1.43%; e.g. one deceased parent). Almost all participants (98.58%) indicated that they were living with one or both parents. Ethnic minority participants were predominantly second‐generation immigrants (74.59%) who were born in Italy. The remaining (25.41%) were first‐generation immigrants, who had been living in Italy for an average of 7.53 years (*SD* = 5.11, range: 6 months−15.50 years) at T1. Most of the first‐generation immigrants (67.74%) were born in other European countries, such as Romania, Ukraine and Albania, while the rest were born in Africa (17.74%), Asia (8.06%) and North, Central and South America (6.46%). Most parents of the second‐generation immigrant adolescents migrated from other European countries (40.56% and 49.45% of fathers and mothers, respectively), with Albanians being the most represented group, while the rest migrated from Africa (20.56% and 19.23% of fathers and mothers, respectively); Asia (2.78% and 2.75% of fathers and mothers, respectively); North, Central and South America (3.89% of fathers, 6.59% of mothers); and the Middle East (1.11% of fathers, 0.55% of the mothers). In terms of reasons for migration, most of the participants indicated their family's economic situation as the main reason (35.25% and 29.51% of fathers and mothers, respectively), family reunification (7.79% and 23.36% of fathers and mothers, respectively), other reasons (e.g. to study, to escape war; 3.69% and 6.97% of fathers and mothers, respectively) or preferred not to answer this question (53.28% and 40.17% of fathers and mothers, respectively).

Most Italian majority (75.95%) and ethnic minority (60.66%) participants in the final longitudinal sample completed all three assessments, while the remaining (24.05% majority and 39.34% ethnic minority) completed two. Within each assessment, missing items (due to both non‐participation in the assessment and non‐response to certain questionnaire items) ranged from 4.19% to 15.00% (majority group) and from 19.67% to 31.97% (ethnic minority group). The Little's (1988) Missing Completely at Random (MCAR) test revealed a normed χ^2^ (χ^2^/df) of 1.19 (majority group) and 1.11 (ethnic minority group), indicating that data were likely missing at random. Missingness within the included sample was further inspected by means of attrition analyses (see Supplemental Materials). Overall, few significant differences emerged according to participation across waves, and those that did emerge were generally small in magnitude and unlikely to have meaningfully affected the study findings. Therefore, all participants in the final longitudinal sample (*N* = 984) were included in the analyses and missing data were handled by means of the Full Information Maximum Likelihood (FIML; Kelloway, 2015) method in M*plus* (Muthén & Muthén, 1998–2017).

### Procedure

This project was approved by the Ethics Committee of the Alma Mater Studiorum University of Bologna (Italy). Permission from school principals was obtained to administer the questionnaire during class hours. After receiving approval from school principals, active parental consent was secured by sending consent forms to both parents at least 1 week prior to the T1 data collection. Then, students were given both oral and written information about the study and asked to assent to the study participation. At each time point, school principals informed teachers about the project and the scheduled data collection through written and digital communications. During the administration of the questionnaires, researchers were present, and teachers could choose to remain in the classroom or leave. Students who did not provide assent or whose parents did not give consent remained in the classroom and were engaged in alternative school activities. Data collection at T1 (May 2019) and T2 (November 2019) was conducted in classrooms using a paper‐and‐pencil questionnaire, while T3 (May 2020) was conducted remotely due to the COVID‐19 pandemic. Each participant generated a unique code in order to link the responses across waves while maintaining confidentiality. Participation was voluntary, with the option to drop out at any time. The study was designed to reach a sample of about 1000 adolescents so that, given the distribution of foreign‐born adolescents in the context under investigation, the ethnic minority subgroup would consist of at least 200 participants, in order to have a suitable subsample for analyses using a structural equation modelling framework (Little, 2013).

### Measures

Participants from the Italian majority and ethnic minority groups reported their socio‐demographic information (e.g. age, sex, nationality) and completed different versions of the remaining measures based on their background. Descriptives and Cronbach's alphas for all measures are reported in Supplementary Table S3.

#### Acculturation preferences in peripheral and central domains

Participants' preferences for different acculturation strategies were measured with the Acculturation Strategies and Attitudes Scale, based on the Relative Acculturation Extended Model (RAEM; Navas et al., 2005; for the Italian version, see Mancini & Bottura, 2014). The version for the Italian majority group included items related to the preference for ethnic minorities' heritage culture maintenance (‘How much should people of foreign origin maintain the traditions of their country of origin in each of the following domains [i.e., school, consumer habits, friendships, family relationships, religion, ways of thinking]?’) and destination culture adoption (‘How much should people of foreign origin adopt the traditions of the destination country [Italy] in each of the following domains [i.e., school, consumer habits, friendships, family relationships, religion, ways of thinking]?’). The version for the ethnic minority group included items related to their heritage culture maintenance (i.e. ‘How much are you currently maintaining the traditions of your country of origin in each of the following domains [i.e., school, consumer habits, friendships, family relationships, religion, ways of thinking]?’) and destination culture adoption (i.e. ‘How much are you currently adopting the traditions of the destination country [Italy] in each of the following domains [i.e., school, consumer habits, friendships, family relationships, religion, ways of thinking]?’). These acculturation preferences were measured with 12 items (6 for maintenance of the culture of origin and 6 for adoption of the destination culture), each scored on a 5‐point Likert‐type scale, ranging from 1 (*not at all*) to 5 (*very much*).

#### Quantity and quality of intergroup contact in school and out‐of‐school contexts

Participants' quantity and quality of intergroup contact experiences were measured with the Intergroup Contact Interactions Scale (ICIS; Karataş, Rubini, et al., 2023). Adolescents from the majority group answered questions about their interactions with individuals of foreign origin, whereas adolescents from the ethnic minority group answered questions about their interactions with Italians. The same items were repeated twice to measure adolescents' contact experiences in school and out‐of‐school contexts separately. The first item measured the quantity of contact (‘In the past six months, have you met and talked with people of foreign origin [Italian people] at school [out‐of‐school]?’). The next 10 items measured the quality of contact experiences, so either positive (5 items; e.g. ‘They have been friendly to you’.) or negative (5 items; e.g. ‘They have been hostile towards you’.). All items assessing quantity and quality of contact were scored on a 5‐point Likert‐type scale ranging from 1 (*never*) to 5 (*very often*).

### Strategy of analyses

Descriptive and preliminary checks were conducted in IBM SPSS for Windows, version 26 (IBM Corp., 2019). All the remaining analyses were conducted in M*plus* 8.11 (Muthén & Muthén, 1998–2017), using Maximum Likelihood Robust (MLR) estimator (Satorra & Bentler, 2001). Analytic codes can be found at: https://osf.io/pcdku.

As a preliminary step, descriptive statistics, correlations, reliabilities and sample attritions analyses were conducted. Further, intraclass correlation coefficients were computed for all study variables to examine how much variance each variable had at the within‐ (i.e. over time) and between‐person levels. To this end, unconditional multilevel models (with repeated observations nested in individuals) were tested separately for the majority and ethnic minority samples. Next, confirmatory factor analyses were conducted to examine the factorial structure of the acculturation preference scales across domains. Further, longitudinal measurement invariance was tested for each study variable, separately for the majority and ethnic minority samples. The full procedures of factorial and invariance checks are detailed in the Supplemental Materials.

To address the goals of this study, Random‐Intercept Cross‐Lagged Panel Models (RI‐CLPM; Hamaker et al., 2015) were estimated to examine the within‐ and between‐person associations between acculturation preferences in central and peripheral domains and quantity and quality of intergroup contact. A total of four models were estimated. Two models focused on the associations between acculturation preferences and intergroup contact in school, separately for majority (Model A) and ethnic minority (Model B) youth, while the other two models examined the longitudinal interplay between acculturation preferences and intergroup contact in the out‐of‐school context for majority (Model C) and ethnic minority (Model D) adolescents. The RI‐CLPM allows for separating the variance of longitudinal observations into stable between‐person differences (i.e. the random intercepts) and within‐person fluctuations over time. Thus, the between‐person level provides information about inter‐individual differences, while the within‐person level sheds light on intra‐individual processes (Hamaker et al., 2015).

At each level, different types of variables and associations can be estimated. At the between‐person level, one random intercept is estimated for each variable, and it represents an individual's average score of said variable across the time occasions. Significant *between‐person correlations* suggest that individuals who score generally high in one variable (e.g. destination culture adoption) also score generally high (or low) on another (e.g. positive contact). At the within‐person level, each variable represents an individual's temporary deviation from their own stable mean (i.e. random intercept) of the same construct. *Within‐person autoregressions* (e.g. the association from within‐person levels of contact quantity at T to contact quantity at T + 1) represent carry‐over effects and indicate the extent to which an individual with elevated levels of a variable at one time point is likely to display a similar fluctuation at the following one (Hamaker et al., 2015). *Within‐person cross‐lagged paths* (e.g. the association from destination culture adoption at T to contact quantity at T + 1) represent the extent to which fluctuations in one variable lead to fluctuations in another variable at the following time. *Within‐person correlations at T1* (e.g. the correlation between destination culture adoption and contact quantity at T1) indicate the extent to which two variables vary together, while *within‐person residual covariances at T2 and T3* (e.g. the correlation between unexplained within‐person levels of destination culture adoption and contact quantity at T2) represent the extent to which a temporary fluctuation from the stable mean in one variable occurs together with a temporary fluctuation in another variable.

All models were estimated adopting a stepwise approach to identify the most parsimonious solution. In the first step unconstrained models (M1) were estimated to assess the within‐person cross‐lagged associations between variables, while controlling for: (a) within‐person stability paths (T1 → T2 and T2 → T3); (b), within‐person correlations (at T1); (c) within‐person residual covariances at T2 and T3; and (d) between‐person correlations. Within‐person cross‐lagged associations of .03, .07 and .12 are indicative of small, medium and large effects, respectively (Orth et al., 2022). In the second step, models (M2) with within‐person autoregressions constrained to be equal across time (i.e. T1 → T2 autoregressions constrained to be equal to their respective T2 → T3 autoregressions) were estimated and compared against the respective M1. In the third step, models (M3) with both within‐person autoregressions and cross‐lagged paths constrained to be equal across time (i.e. T1 → T2 cross‐lagged paths constrained to be equal to their respective T2 → T3 cross‐lagged paths) were estimated and compared against the respective M2. In the final step, models (M4) with within‐person autoregressions, cross‐lagged paths and residual covariances at T2 and T3 constrained to be equal across time (i.e. T2 residual covariances constrained to be equal to their respective T3 covariances) were estimated and compared against M3. Each step allows to establish whether associations at the within‐person level are invariant (i.e. equal) over time, confirming assumptions of stationarity of developmental processes and leading to a more parsimonious representation of findings. To assess such invariance, each model is evaluated independently based on a combination of fit indices: the Comparative Fit Index (CFI) with values above .90 and .95 indicative of acceptable and excellent fit; the Standardized Root Mean Square Residual (SRMR) and the Root Mean Square Error of Approximation (RMSEA) with values lower than .08 and .05 indicative of acceptable and excellent fit, respectively (Byrne, 2012); and the 90% confidence interval of the RMSEA with an upper bound value lower than .10 indicating acceptable fit (Chen et al., 2008). Further, nested models (i.e. M1 vs. M2, M2 vs. M3 and M3 vs. M4) were compared against each other. When at least two of the following criteria are satisfied, this would indicate that full invariance of associations is not established: a significant Δχ^2^_SB_ (Satorra & Bentler, 2001), ΔCFI ≥ − .010 and ΔRMSEA ≥ .015 (Chen et al., 2008). If full invariance could not be established, the equality of paths across time points was inspected by means of pairwise Wald test statistics. Paths that significantly differed from each other over time were then released to reach partial invariance of within‐person associations. The final best fitting and most parsimonious model was retained.

## RESULTS

### Preliminary analyses

Intraclass correlation coefficients are reported in Table S3. For the majority group, slightly less than half of the variance was at the between‐person level for most variables (ranging from 34% for destination culture adoption in peripheral domains to 46.7% for quantity of contact in the out‐of‐school context), while for positive contact in school variance was equally divided at the between‐ (50.4%) and within‐person levels (49.6%). For the ethnic minority group, a small portion of variance in quantity of contact in school (20.8%) and negative contact in the out‐of‐school context (23.3%) was at the between‐person level, while for the remaining variables slightly less than half (from 34.3% in negative contact at school to 42.7% in adoption in central domains) or roughly equal variance (50.1% for heritage culture maintenance in central domains) was at the between‐person level.

Correlations among study variables are reported in Table S4, separately for majority and ethnic minority participants. Results of confirmatory factor analyses (see Supplementary Table S5) showed that, for both heritage culture maintenance and destination culture adoption, two‐factor models distinguishing acculturation preferences in central and peripheral domains provided a better fit compared to one‐factor models aggregating preferences across domains. These solutions were thus retained for subsequent analyses. Partial scalar measurement invariance was established for ethnic minority adolescents' destination culture adoption and negative contact out‐of‐school, whereas all the remaining acculturation and contact quality measures for both majority and ethnic minority youth showed full scalar invariance (see Supplementary Table S6).

### Acculturation and contact in school

Random‐intercept cross‐lagged panel models examining the longitudinal interplay of acculturation preferences (in central and peripheral domains) and quantity and quality of intergroup contact in school were tested separately for majority (Model A) and ethnic minority (Model B) adolescents. All models displayed a very good fit (Table 1). For the majority group full invariance of within‐person associations was reached, while for the ethnic minority group full invariance of within‐person autoregression and cross‐lagged paths, and partial invariance of residual covariances, was established.

**TABLE 1:** Random intercept cross‐lagged panel models: Model fit and comparisons.

Models	Model fit					Model comparisons			
	χ_SB_^2^	df	CFI	SRMR	RMSEA [90% CI]	Models	Δχ_SB_^2^	ΔCFI	ΔRMSEA
Model A: Majority's acculturation preferences and contact in school									
Unconstrained (M1)	29.457	21	.998	.016	.023 [.000, .042]				
Autoregressions fixed (M2)	49.853	28	.994	.023	.032 [.017, .047]	M2‐M1	19.441 (7)**	−.004	.009
Autoregressions and cross‐lagged paths fixed (M3)	106.289	70	.990	.036	.026 [.015, .036]	M3‐M2	57.091 (42)	−.004	−.006
Autoregressions, cross‐lagged paths and residual covariances fixed (M4)	145.398	91	.985	.042	.028 [.019, .037]	M4‐M3	38.369 (21)*	−.005	.002
Model B: Ethnic minority's acculturation preferences and contact in school									
Unconstrained (M1)	27.117	21	.996	.021	.035 [.000, .069]				
Autoregressions fixed (M2)	43.472	28	.989	.026	.048 [.015, .075]	M2‐M1	16.733 (7)*	−.007	.013
Autoregressions and cross‐lagged paths fixed (M3)	86.450	70	.988	.048	.031 [.000, .051]	M3‐M2	44.092 (42)	−.001	−.017
Autoregressions, cross‐lagged paths and residual covariances fixed (M4)	134.471	91	.968	.083	.045 [.027, .060]	M4‐M3	42.252 (21)**	−.020	.014
Autoregressions, cross‐lagged paths and residual covariances partially fixed (M4a)^1^	111.170	86	.981	.061	.035 [.011, .053]	M4a‐M3	23.911 (16)	−.007	.004
Model C: Majority's acculturation preferences and contact out‐of‐school									
Unconstrained (M1)	10.406	21	1.000	.009	.000 [.000, .000]				
Autoregressions fixed (M2)	38.072	28	.997	.018	.022 [.000, .038]	M2‐M1	34.055 (7)***	−.003	.022
Autoregressions partially fixed (M2a)^2^	21.757	26	1.000	.013	.000 [.000, .023]	M2a‐M1	12.554 (5)*	.000	.000
Autoregressions and cross‐lagged paths fixed (M3)	87.289	68	.995	.031	.020 [.000, .031]	M3‐M2a	64.592 (42)*	−.005	.020
Autoregressions and cross‐lagged paths partially fixed (M3a)^3^	74.361	67	.998	.029	.012 [.000, .026]	M3a‐M2a	51.902 (41)	−.002	.012
Autoregressions, cross‐lagged paths and residual covariances fixed (M4)	98.289	88	.997	.035	.013 [.000, .024]	M4‐M3a	23.875 (21)	−.001	.001
Model D: Ethnic minority's acculturation preferences and contact out‐of‐school									
Unconstrained (M1)	47.160	21	.979	.037	.072 [.044, .099]				
Autoregressions fixed (M2)	51.703	28	.981	.043	.059 [.033, .084]	M2‐M1	8.104 (7)	.002	−.013
Autoregressions and cross‐lagged paths fixed (M3)	83.098	70	.990	.072	.028 [.000, .049]	M3‐M2	37.348 (42)	.009	−.031
Autoregressions, cross‐lagged paths and residual covariances fixed (M4)	123.254	91	.974	.079	.038 [.018, .055]	M4‐M3	38.755 (21)*	−.016	.010
Autoregressions, cross‐lagged paths and residual covariances partially fixed (M4a)^4^	106.338	88	.985	.075	.029 [.000, .048]	M4a‐M3	23.118 (18)	−.005	.001

Abbreviations: CFI, Comparative Fit Index; CI, Confidence Interval; df, Degree of Freedom; M, Model; RMSEA, Root Mean Square Error of Approximation; SRMR, Standardized Root Mean Square Residual; Δ, Change in the parameter; χSB^2^, Satorra & Bentler's corrected Chi‐square.

^1^ In this model, the following residual covariances were freed: between maintenance and adoption in central domains, between maintenance in central domains and negative contact, between quantity, positive and negative contact.

^2^ In this model, the autoregression paths of adoption in central domains and contact quantity were freed.

^3^ In this model, the cross‐lagged path from positive contact to contact quantity was freed.

^4^ In this model, the following residual covariances were freed: between maintenance and adoption in central domains, between quantity and positive contact, and between positive and negative contact.

* *p* < .05;

** *p* < .01;

*** *p* < .001.

#### Majority group

**FIGURE 1:**
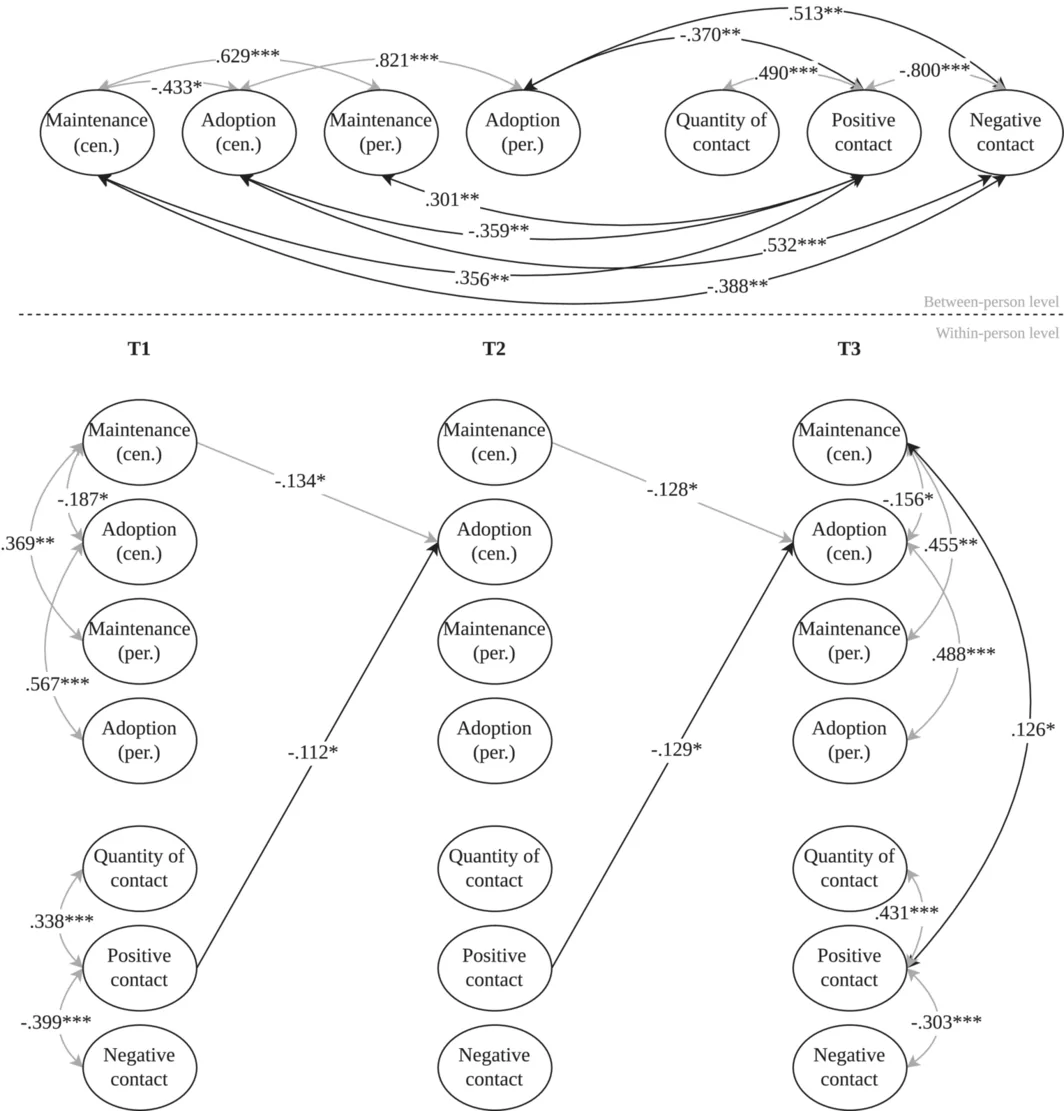
Significant standardized results of the random‐intercept cross‐lagged model with acculturation preferences and contact in school for the majority group. For the sake of clarity, only significant within‐ and between‐person level associations are displayed. Gray continuous arrows indicate within‐construct effects. Cen., central domains; per., peripheral domains; T, Time. ^*^ *p* < .05, ^**^ *p* < .01, ^***^ *p* < .001.

Results (Figure 1, Table S7) highlighted several associations between majority's acculturation preferences and quality, but not quantity, of contact at two levels of analysis. At the *between‐person level*, majority adolescents who showed a generally higher preference for heritage culture maintenance in central and, to a lesser extent, peripheral domains engaged in more positive and less negative intergroup contact at school. Conversely, those who more strongly preferred destination culture adoption in both domains reported, on average, less positive and more negative contact. At the *within‐person level*, only one significant cross‐lagged association emerged, from positive contact to preference for adoption. Specifically, majority adolescents who engaged in more positive contact compared to their general mean reported a decreased preference for destination culture adoption in central domains at the following time point. Albeit statistically significant, this association did not differ in magnitude from its not significant reversed one (i.e. from destination culture adoption in central domains to positive contact) based on Wald tests (Wald = 3.35, *p* = .067). While acculturation preferences and contact in school did not significantly covary at T1, at later time points temporary increases in preference for heritage culture maintenance occurred with concurrent increases in positive contact in school.

#### Ethnic minority group

**FIGURE 2:**
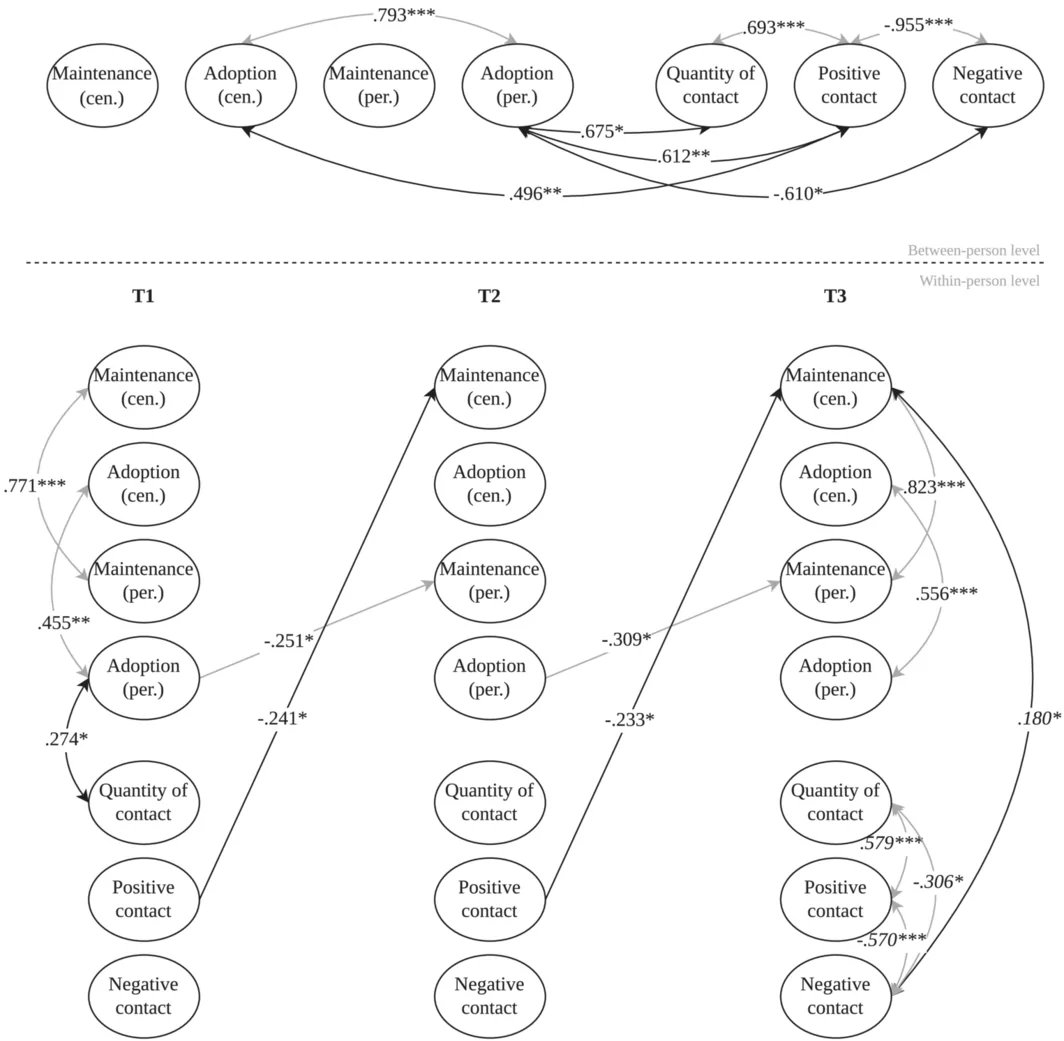
Significant standardized results of the random‐intercept cross‐lagged model with acculturation preferences and contact in school for the ethnic minority group. For the sake of clarity, only significant within‐ and between‐person level associations are displayed. Gray continuous arrows indicate within‐construct effects. Cen., central domains; per., peripheral domains; T, Time. ^*^ *p* < .05, ^**^ *p* < .01, ^***^ *p* < .001.

Results (Figure 2, Table S7) revealed only a few associations between ethnic minority adolescents' acculturation preferences and contact in school. At the *between‐person level*, ethnic minority adolescents who generally adopted the destination culture to a higher extent also engaged more frequently in intergroup contact and reported, on average, more positive and less negative intergroup encounters. At the *within‐person level*, temporary increases in the frequency of positive contact experiences from adolescents' average were associated with subsequent decreases in their preference for heritage culture maintenance in central domains at the subsequent time point. This path significantly differed from its reversed one (i.e. from heritage culture maintenance to positive contact) which did not reach statistical significance (Wald = 4.24, *p* = .039). Similar to between‐person level associations, ethnic minority youth who preferred to adopt the destination culture in peripheral domains at T1 also reported more frequent intergroup contact concurrently. Moreover, at T3 only, temporary increases in adolescents' preference for heritage culture maintenance in central domains occurred together with increases in negative contact experiences in school.

### Acculturation preferences and contact out‐of‐school

Random‐intercept cross‐lagged panel models examining the interplay between acculturation preferences and contact in the out‐of‐school context were tested separately for majority (Model C) and ethnic minority groups (Model D). These models had a good fit (Table 1). Partial invariance of autoregression and cross‐lagged paths and full invariance of residual covariances was reached for the majority group, while full invariance of autoregressions and cross‐lagged paths and partial invariance of residual covariances was found for the ethnic minority.

#### Majority group

**FIGURE 3:**
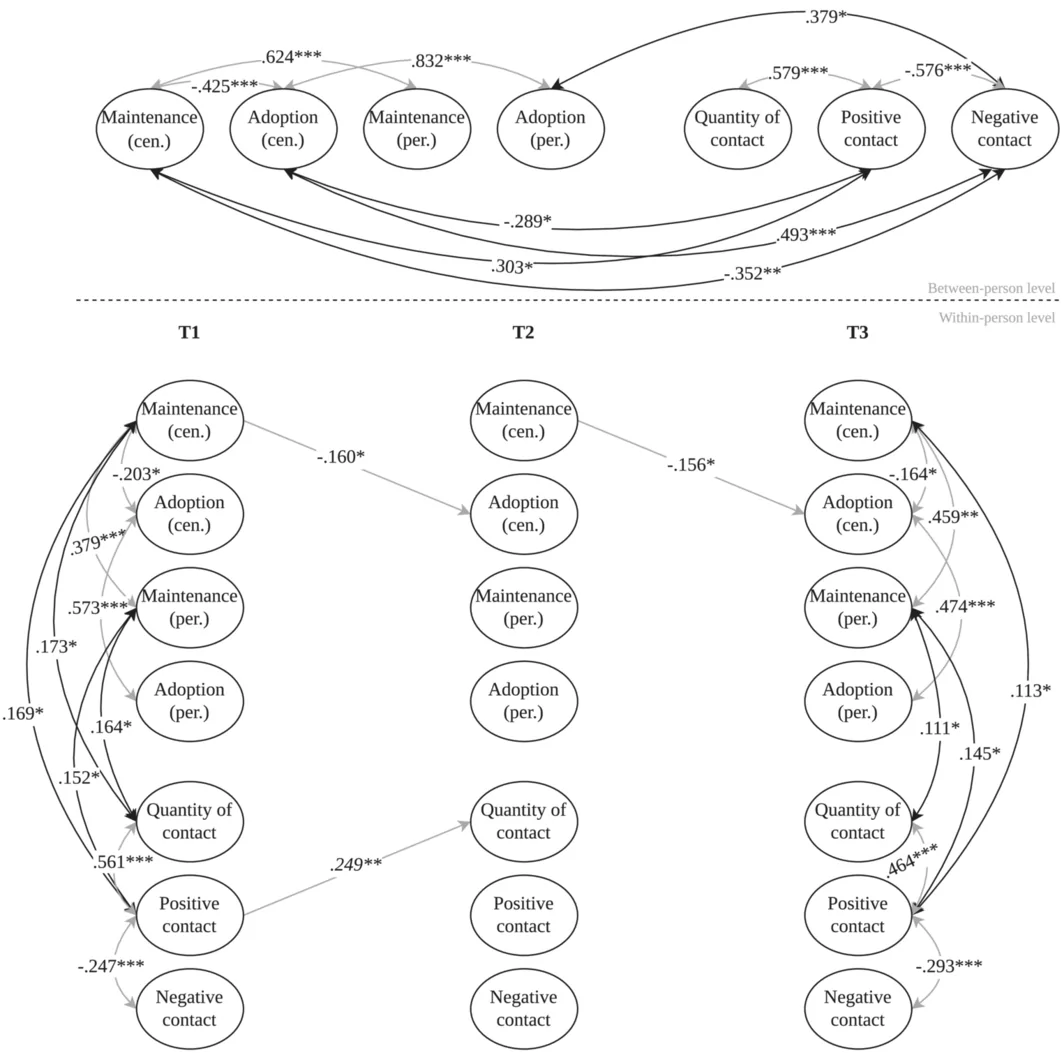
Significant standardized results of the random‐intercept cross‐lagged model with acculturation preferences and contact out‐of‐school for the majority group. For the sake of clarity, only significant within‐ and between‐person level associations are displayed. Gray continuous arrows indicate within‐construct effects. Cen., central domains; per., peripheral domains; T, Time. ^*^ *p* < .05, ^**^ *p* < .01, ^***^ *p* < .001.

Results (Figure 3, Table S8) highlighted the presence of concurrent, but not longitudinal associations between majority adolescents' acculturation preferences and quantity and quality of contact in the out‐of‐school context. At the *between‐person level*, adolescents who, on average, had a higher preference for heritage culture maintenance in the central domain reported more positive and less negative contact, while those with a higher preference for adoption of destination culture reported generally less positive and more negative intergroup encounters out‐of‐school. Similarly, at the *within‐person level*, adolescents with a higher preference for heritage culture maintenance in both central and peripheral domains reported more frequent and more positive contact at T1, and increases in preference for maintenance at T2 and T3 co‐occurred with increases in both contact quantity and positive contact.

#### Ethnic minority group

**FIGURE 4:**
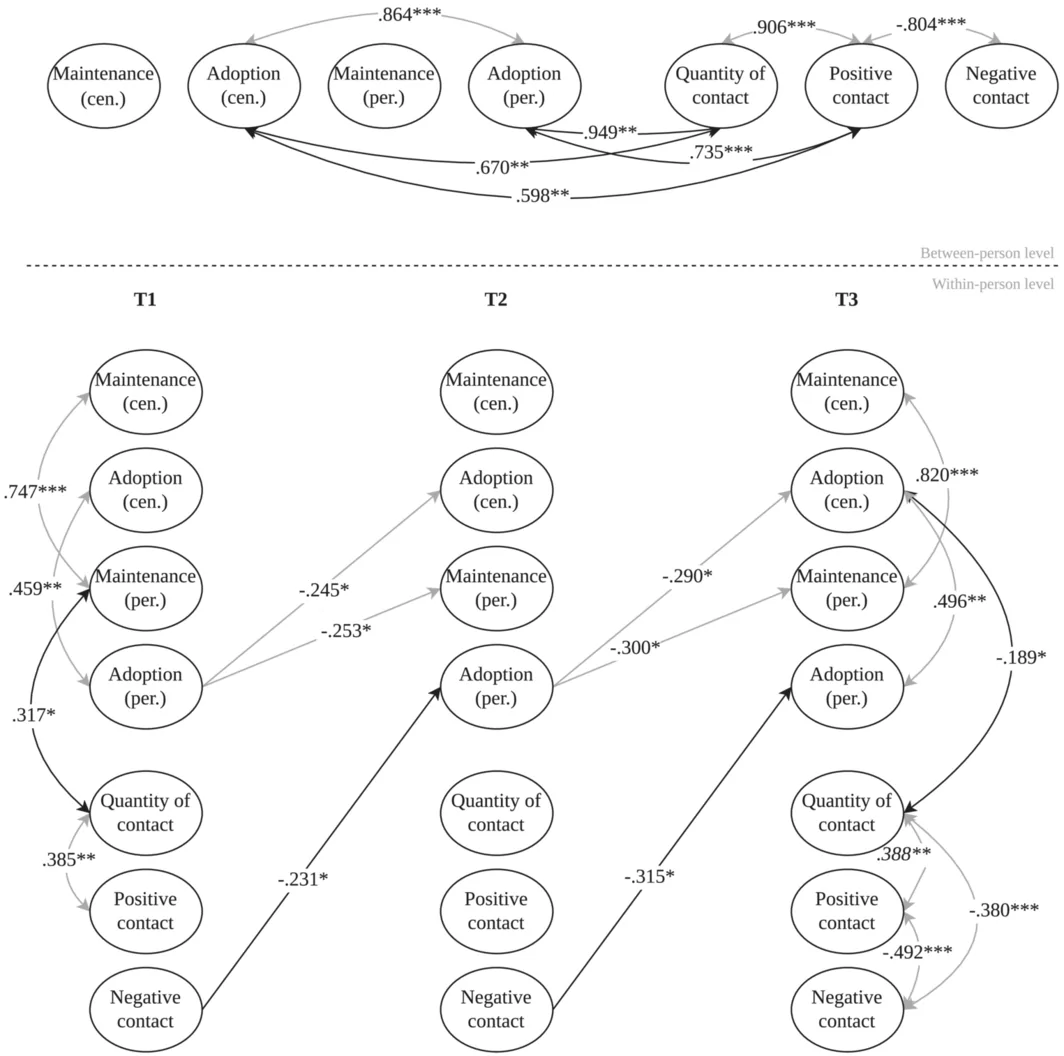
Significant standardized results of the random‐intercept cross‐lagged model with acculturation preferences and contact out‐of‐school for the ethnic minority group. For the sake of clarity, only significant within‐ and between‐person level associations are displayed. Gray continuous arrows indicate within‐construct effects. Cen., central domains; per., peripheral domains; T, Time. ^*^ *p* < .05, **p < .01, ***p < .001.

Results (Figure 4, Table S8) indicated both concurrent and longitudinal associations between ethnic minority adolescents' acculturation preferences and the quantity and quality of contact out‐of‐school. At the *between‐person level*, a higher general preference for destination culture adoption in both central and peripheral domains was associated with more frequent and more positive contact experiences. However, at the *within‐person level*, preference for heritage culture maintenance and contact quantity were positively correlated at T1, and temporary increases at T3 in adolescents' preference for adoption in central domains co‐occurred with decreases in the frequency of contact. Furthermore, longitudinally, adolescents who experienced a temporary increase in negative contact out‐of‐school at one time point reported a decreased preference for destination culture adoption in peripheral domains at the subsequent time point. Of note, this longitudinal path did not differ in magnitude from the reversed pathway (i.e. from destination culture adoption in peripheral domains to negative contact) which was not statistically significant (Wald = 1.20, *p* = .274).

### Sensitivity analyses

As a sensitivity check, the four RI‐CLPM models were tested again using the Type = Complex function in M*plus*, which provides robust estimates accounting for the nested structure of the data (i.e. students nested in classrooms). Detailed procedures and results of sensitivity models are available in Supplemental Materials (Tables S9–S11). Results largely replicated the main findings with only a few exceptions in the models examining the longitudinal associations between acculturation preferences and contact in the out‐of‐school context. Nevertheless, these differences were marginal and did not involve associations between acculturation and contact, confirming the robustness of current findings.

## DISCUSSION

Adolescence, as a crucial period for developing social attitudes (Cook & Bird, 2011), represents a unique opportunity to examine acculturation tendencies and intergroup relations among majority (receiving society) and ethnic minority group members (Berry, 2005) in current multicultural societies. Despite this relevance, existing research has examined the link between acculturation and intergroup contact among adolescents primarily using cross‐sectional designs, often framing contact conceptually as an antecedent of acculturation, with a more recent limited number of studies employing longitudinal approaches (e.g. González et al., 2017; Hässler et al., 2018). The current study extends this work by examining the longitudinal associations between acculturation preferences and intergroup contact quality and quantity among both majority and ethnic minority adolescents over time.

### Group differences in the interplay between acculturation preferences and intergroup contact

#### Majority group

Present longitudinal findings indicate that, among majority group adolescents, acculturation preferences and intergroup contact were associated primarily at the level of stable between‐person differences rather than dynamic within‐person change. Specifically, at the *between‐person level*, adolescents who generally reported a higher preference for cultural heritage maintenance, especially in central domains, also reported higher positive (supporting Hypothesis 1a) and lower negative (supporting Hypothesis 1b) intergroup contact in both school and out‐of‐school contexts. Moreover, majority adolescents who generally showed a higher preference for destination culture adoption in both domains also reported lower positive (consistent with Hypothesis 2a) and higher negative (supporting Hypothesis 2b) contact across both contexts. With respect to Hypothesis 3, which proposed that majority adolescents' preference for heritage culture maintenance would be more strongly associated with intergroup contact quantity in school than out‐of‐school, the results did not provide support. Preferences for heritage culture maintenance were not systematically related to contact quantity in either context.

At the *within‐person level*, limited longitudinal associations were observed. Specifically, majority adolescents who engaged in more positive intergroup contact in school compared to their general mean reported a decreased preference for destination culture adoption in central domains at the subsequent time point. This finding did not support Hypothesis 4, which expected majority adolescents' acculturation preferences to show stronger longitudinal associations with subsequent intergroup contact than the reverse pattern.

Taken together, these findings indicate that associations between acculturation preferences and intergroup contact among majority adolescents emerged primarily at the between‐person level. Adolescents who generally endorsed heritage culture maintenance reported more favourable contact experiences, whereas those who generally endorsed destination culture adoption reported less favourable contact experiences. This pattern suggests that majority adolescents' acculturation preferences may function as relatively stable orientations towards cultural diversity and intergroup relations, in line with acculturation frameworks (Berry, 2005; Bourhis et al., 1997). Rather than being strongly linked to changes in contact experiences over time, these preferences appear to be associated with how majority adolescents generally experience and evaluate intergroup interactions (Brown & Hewstone, 2005).

#### Ethnic minority group

Fewer, but more context‐specific, between‐person associations emerged for ethnic minority adolescents, alongside stronger evidence at the within‐person level. At the *between‐person level*, heritage culture maintenance preferences in central and peripheral domains were not consistently associated with positive (not supporting Hypothesis 5a) or negative contact (not supporting Hypothesis 5b) in either context. However, at the *within‐person level*, temporary increases in heritage culture maintenance preferences in central domains co‐occurred concurrently with increases in negative contact in school, providing partial support for Hypothesis 5b. In contrast, ethnic minority adolescents who generally reported a higher preference for destination culture adoption reported higher contact quantity and positive contact (supporting Hypothesis 6a), as well as lower negative contact (consistent with Hypothesis 6b), particularly in the out‐of‐school context and across both domains.

At the *within‐person level*, intergroup contact quantity and quality showed stronger associations with subsequent changes in ethnic minority adolescents' acculturation preferences than the reverse pattern (supporting Hypothesis 7). Specifically, ethnic minority adolescents who experienced more positive intergroup contact in school compared to their general mean reported a decreased preference for heritage culture maintenance in central domains at the subsequent time point. Similarly, adolescents who experienced a temporary increase in negative intergroup contact out‐of‐school reported a decreased preference for destination culture adoption, particularly in peripheral domains, at the subsequent time point. In addition, concurrent within‐person associations indicated that temporary increases in preferences for heritage culture maintenance in both central and peripheral domains occurred together with increases in intergroup contact quantity at earlier measurement occasions. In contrast, at later measurement occasions, temporary increases in destination culture adoption preferences in central domains co‐occurred with decreases in intergroup contact quantity out‐of‐school.

This evidence suggests that acculturation preferences among ethnic minority adolescents appeared to be more closely tied to fluctuations in intergroup experiences than was the case for majority adolescents. The stronger within‐person associations observed for this group point to a more dynamic adjustment process, in which acculturation preferences were associated with preceding contact experiences. Although this pattern may initially seem at odds with theory suggesting that ethnic minority members' acculturation preferences are more strongly rooted in internal motivations (Zagefka et al., 2014), developmental perspectives emphasize adolescents' heightened sensitivity to social relationships, peer evaluation and group norms (Hjerm et al., 2018; Karataş, 2024; Merrilees et al., 2023). Ethnic minority adolescents' acculturation preferences may therefore be particularly responsive to everyday interactions with majority peers (Motti‐Stefanidi & Masten, 2017). Positive intergroup contact may signal social acceptance and belonging, thereby reducing the salience of heritage culture maintenance, whereas negative contact may undermine motivation to adopt the destination culture. These findings are broadly consistent with research indicating that orientation towards the receiving society is often linked to greater opportunities for positive intergroup engagement (Berry, 1997; Vedder & Phinney, 2014).

Overall, these group‐specific patterns indicate that links between acculturation preferences and intergroup contact unfold through different processes for majority and ethnic minority adolescents. Rather than reflecting a uniform pattern, the acculturation‐contact link appears to be shaped by adolescents' structural position within intergroup relations, and the meaning acculturation preferences hold for each group. These differences point to the importance of examining the directionality of associations between acculturation preferences and intergroup contact over time.

### Directionality of acculturation‐contact associations

A central aim of the present study was to examine whether acculturation preferences function as antecedents of intergroup contact patterns, or whether intergroup contact is more consistently associated with subsequent changes in acculturation preferences over time. Across both groups, the findings provided stronger evidence for the latter pattern, especially at the within‐person level. While most associations between acculturation preferences and contact emerged as stable between‐person differences, the longitudinal within‐person effects that did emerge more often indicated that contact experiences were associated with subsequent changes in acculturation preferences than vice versa.

This pattern may be understood in light of the developmental characteristics of adolescence. Intergroup contact constitutes a concrete and emotionally salient social experience, whereas acculturation preferences involve more abstract beliefs about cultural principles (Laursen & Veenstra, 2021) and whether cultural heritage should be maintained or the destination culture adopted. At the same time, the ability to engage in mature hypothetical and inferential thinking continues to develop throughout adolescence (Kuhn, 2009). As a result, adolescents might find it easier to adjust their acculturation preferences in response to meaningful intergroup experiences than to translate pre‐existing acculturation preferences into changes in everyday behaviour.

Contextual differences may further contribute to this pattern. In school contexts, intergroup contact opportunities are structured, recurrent and institutionally constrained, leaving adolescents with limited autonomy over whether and how often contact occurs (Thijs & Verkuyten, 2014). Under such conditions, acculturation preferences may have relatively limited opportunity to influence contact quantity. In contrast, out‐of‐school contexts provide greater scope for agency and choice, making contact experiences in these settings particularly relevant for adolescents' subsequent acculturation and intergroup relations.

Additionally, aspects of study design, including the length of measurement intervals and the age group studied, may have further contributed to this pattern and are discussed in the Limitations section. More broadly, these findings suggest that acculturation preferences may be more closely linked to how adolescents interpret and evaluate intergroup encounters than to whether contact occurs in the first place. Although evidence for acculturation preferences predicting later contact was limited, stable preferences for heritage culture maintenance and destination culture adoption were consistently associated with the quality of intergroup contact at the between‐person level. This pattern suggests that acculturation preferences may function less as direct antecedents of contact opportunities and more as orientations through which intergroup experiences are understood and evaluated. Together, these findings underscore the value of considering both developmental and contextual influences when examining the relationship between acculturation and intergroup contact during adolescence.

### Practical implications

The present findings highlight that rather than reflecting a uniform process, the links between acculturation preferences and intergroup contact vary across majority and ethnic minority adolescents and across social settings, suggesting that one‐size‐fits‐all approaches may have limited effectiveness.

For majority adolescents, the findings underscore the importance of fostering inclusive norms and positive intercultural climates across schools and other youth environments. Schools are particularly well positioned to promote positive intergroup relations because they provide regular opportunities for interaction and can establish norms that support inclusion, respect and cultural diversity (Maratia et al., 2025). Creating settings in which cultural diversity is recognized and valued may contribute to more positive intergroup experiences and reduce the likelihood that cultural differences become sources of tension or exclusion.

For ethnic minority adolescents, the evidence suggests that everyday intergroup experiences both in and out‐of‐school contexts may play a role in shaping acculturation preferences. Given the role of both of these contexts in adolescents' lives, efforts to promote positive intergroup relations may benefit from extending beyond formal educational settings to include community‐based activities and other informal spaces for intercultural engagement (Guèvremont et al., 2014). Overall, these findings emphasize that intergroup contact and acculturation are interconnected and dynamic processes shaped by social context. Promoting positive intergroup relations during adolescence may therefore require coordinated efforts across multiple settings that reflect the differing realities of majority and ethnic minority adolescents.

### Limitations and future directions

First, this study relied on state‐of‐the‐art longitudinal models (i.e. RI‐CLPM) to examine within‐ and between‐person associations between acculturation and intergroup contact in a large sample of adolescents, and accounted for the multifaceted nature of these phenomena. Although no convergence nor estimation issue emerged in the current study, RI‐CLPMs typically require large sample sizes to detect small to medium effects (Mulder, 2023). Therefore, findings from the current study, particularly for the ethnic minority group, should be further supported by additional research with larger representative samples of adolescents.

Second, due to conceptual differences in the acculturation measures used for majority and ethnic minority adolescents, along with the complexity of the longitudinal models, direct statistical comparisons between groups were not conducted. Further research with larger samples and conceptually comparable measures could directly examine group differences.

Third, the present study focused on adolescents' individual acculturation preferences and did not examine the extent to which majority group expectations and ethnic minority group preferences were aligned or mismatched. Future research could directly examine whether the degree of alignment between majority expectations and minority preferences shapes the quality and quantity of intergroup contact over time.

Fourth, the strength of within‐person associations may further depend on aspects of study design, including the length of measurement intervals and the age group studied (Hamaker, 2023). The dynamics linking intergroup contact and acculturation preferences may unfold at timescales that do not align with the six‐month intervals used in our panel design. Effects operating at shorter timescales may have occurred and dissipated between assessments, whereas effects requiring longer periods to accumulate may not have been detectable within this timeframe. Moreover, the three‐wave design provides only a relatively coarse representation of developmental processes, potentially limiting the detection of transient within‐person effects. These design considerations may therefore partly account for the predominantly between‐person associations observed in the present study. Future studies employing longitudinal designs with varying assessment intervals could provide a more fine‐grained understanding of how contact experiences and acculturation preferences mutually influence one another over time.

Fifth, the third wave of data collection took place in May 2020, coinciding with the initial outbreak of the COVID‐19 pandemic. During this period, schools were closed and opportunities for face‐to‐face intergroup contact were substantially restricted both inside and outside school settings. Contact patterns reported at T3 may therefore not reflect typical intergroup experiences, and the change in administration format (from paper‐and‐pencil to online) may have introduced additional measurement variability. The T2 → T3 associations should accordingly be interpreted with caution.

Sixth, the current study measured the quality and quantity of contact. This approach may not fully capture the complexity of how adolescents seek, experience and sustain intergroup interactions. Future research could employ more nuanced measures of contact (i.e. intimate, superficial, voluntary), as well as incorporate personal and psychological factors (i.e. intergroup anxiety, empathy or social identity), in order to gain deeper insight into how and why adolescents engage in intergroup contact and how these experiences are shaped by and shape acculturation preferences (Paolini et al., 2018).

Seventh, given that adolescents spend substantial time in close proximity with their classmates, negative interactions in schools may reflect both intergroup processes as well as general peer conflict (Rubin et al., 2006). In this study, negative contact cannot be fully disentangled from non‐intergroup peer conflict. Although descriptive patterns showed higher levels of negative contact in school than out‐of‐school among ethnic minority adolescents, future research could employ direct measures of general peer conflict to disentangle group‐based negative experiences from more general peer conflict in the school context.

Eighth, this study's findings are shaped by its specific context in terms of population and immigration history, limiting generalizability. Specifically, the study was conducted in Italy, which is a key destination for international migrants (Istituto Nazionale di Statistica, 2025). Contexts with different migration histories may vary in their institutional norms, endorsement of multicultural policies and the relative size of ethnic minority groups, possibly altering the salience of acculturation preferences and opportunities for intergroup contact (Bourhis et al., 1997; López‐Rodríguez et al., 2014; Maratia et al., 2026). Future longitudinal research could therefore examine whether these patterns emerge among different populations and societies with different migration histories.

Finally, in the present study, acculturation preferences of the majority and ethnic minority for the ethnic minority group were examined. Therefore, future research could examine acculturation preferences and perceptions in tandem, as this can reveal whether there are alignments or mismatches between perceptions of the majority regarding what the minority wants to do and actual preferences of the majority. The same approach can be applied to the ethnic minority group, meaning how their perceptions of what the majority wants them to do align or mismatch with their own preferences for acculturation. These alignments or mismatches might impact the subsequent intergroup encounters among both majority and ethnic minority individuals (Zagefka et al., 2023). In line with recent research highlighting the gap in the literature on majority group members' preferences regarding their own acculturation (Kunst, 2025), future research could examine how majority adolescents' acculturation preferences for themselves interact with expectations of ethnic minority acculturation in shaping intergroup contact experiences and motivational engagement with contact.

## CONCLUSION

In current multicultural societies, majority and ethnic minority adolescents navigate acculturation and intergroup relations as part of their everyday social experiences across multiple contexts. This longitudinal study highlighted the dynamic interplay between adolescents' acculturation preferences and intergroup contact across school and out‐of‐school contexts, revealing important group and context‐specific differences. The findings indicate that acculturation preferences play a more central role in structuring intergroup contact patterns for majority adolescents, primarily at the between‐person level, but with no evidence of within‐person effects. In contrast, intergroup contact experiences are more closely linked to subsequent changes in acculturation preferences among ethnic minority adolescents, particularly at the within‐person level. Highlighting these distinct patterns, this study points to the importance of building inclusive environments, both within and beyond schools, that foster preferences of integration and positive intergroup relations between majority and minority adolescents.

## AUTHOR CONTRIBUTIONS

**Lucia Jochym Illy:** Writing – original draft; writing – review and editing; formal analysis; software; visualization. **Beatrice Bobba:** Methodology; validation; writing – review and editing; software; formal analysis; visualization; data curation. **Francesca Prati:** Conceptualization; writing – original draft; writing – review and editing; funding acquisition; supervision. **Monica Rubini:** Writing – review and editing; supervision. **Elisabetta Crocetti:** Conceptualization; investigation; funding acquisition; writing – review and editing; methodology; supervision; resources; project administration.

## CONFLICT OF INTEREST STATEMENT

There are no conflicts of interest to disclose.

## Supporting information


**Table S1.** Results of Chi‐square analyses.
**Table S2.** Results of univariate ANOVAs.
**Table S3.** Means (M), Standard Deviations (SDs), Cronbach's Alpha (α) and Intraclass Correlation Coefficients (ICC) of Study Variables.
**Table S4.** Correlations among Study Variables for Italian Majority (above the diagonal) and Ethnic Minority (below the diagonal) Participants.
**Table S5.** Confirmatory Factor Analysis: Model Fit.
**Table S6.** Longitudinal Measurement Invariance Tests for Each Study Measure.
**Table S7.** Standardized Results of RI‐CLPMs with Acculturation Preferences and Contact in School.
**Table S8.** Standardized Results of RI‐CLPMs with Acculturation Preferences and Contact Out‐of‐School.
**Table S9.** Random‐Intercept Cross‐Lagged Panel Models with Type = Complex: Model fit and comparisons.
**Table S10.** Standardized Results of RI‐CLPMs with Type = Complex: Acculturation Preferences and Contact in School.
**Table S11.** Standardized Results of RI‐CLPMs with Type = Complex: Acculturation Preferences and Contact Out‐of‐School.

## Data Availability

Analyses codes and outputs can be accessed on the Open Science Framework page of the project: https://osf.io/pcdku. The datasets generated and/or analysed during the current study are available from the corresponding author on reasonable request.

## Footnotes

^1^
Regarding prior knowledge, only the last author has general knowledge of the data used in the current study as PI of the larger project.

^2^
Sample attrition analyses comparing adolescents in the final longitudinal sample with those that were excluded due to participation in only one wave are available in the Supplemental Materials.
