# Supplementary material for: The longitudinal interplay between acculturation and intergroup contact in adolescence
Source: Br J Soc Psychol. 2026 Sep 2;65(4):e70129. doi: 10.1111/bjso.70129 (PMC13536516; doi:10.1111/bjso.70129)
Supplement: Supplementary file 1 [file BJSO-65-0-s001.docx]

**Supplementary Information for:**

The Longitudinal Interplay Between Acculturation and Intergroup Contact in Adolescence

Table of Contents

[Sample Attrition Analyses – Included *vs*. Excluded Participants	3](#_Toc235117940)

[Majority Group Comparisons	3](#_Toc235117941)

[Ethnic Minority Group Comparisons	5](#_Toc235117942)

[Attrition Within the Final Longitudinal Sample	7](#_Toc235117943)

[Majority Group Comparisons	7](#_Toc235117944)

[Ethnic Minority Group Comparisons	8](#_Toc235117945)

[Table S1	9](#_Toc235117946)

[Table S2	10](#_Toc235117947)

[Descriptives and Correlations among Study Variables	11](#_Toc235117948)

[Table S3	11](#_Toc235117949)

[Table S4	12](#_Toc235117950)

[Confirmatory Factor Analysis	15](#_Toc235117951)

[Table S5	16](#_Toc235117952)

[Longitudinal Measurement Invariance Tests	17](#_Toc235117953)

[Table S6	19](#_Toc235117954)

[Random Intercept Cross-Lagged Panel Models: Full Standardized Results	21](#_Toc235117955)

[Table S7	21](#_Toc235117956)

[Table S8	25](#_Toc235117957)

[Sensitivity Analyses: Random Intercept Cross-Lagged Panel Models with *Type=Complex*	29](#_Toc235117958)

[Table S9	31](#_Toc235117959)

[Table S10	33](#_Toc235117960)

[Table S11	37](#_Toc235117961)

# Sample Attrition Analyses – Included *vs*. Excluded Participants

A total of 1,295 ethnic majority (*n* = 931) and ethnic minority (*n* = 364) adolescents agreed to participate in the “Developing Inclusive Identities in Adolescence” project. Of this total sample of adolescents, 710 participated in all three waves, 275 participated in two waves, and 310 participated only in one wave. The final longitudinal sample of the project has been generated by considering the adolescents who participated in at least two waves of data collection. Additionally, one participant was excluded as (s)he was the only student in one of the classes. As a result, this sample included 984 adolescents (76% of the total number of participants), of which 740 ethnic majority and 244 ethnic minority adolescents.

Sample attrition analyses were performed to compare included and excluded participants. To this end, a series of univariate ANOVAs were conducted to examine whether participants included in the final longitudinal sample significantly differ from the excluded ones on the main study variables (i.e., acculturation and intergroup contact). When significant differences were detected, Cohen’s *d*s were computed to ascertain the magnitude of these effects. These checks were conducted separately for majority and ethnic minority adolescents.

## Majority Group Comparisons

At T1, majority adolescents in the final longitudinal sample did not significantly differ from the excluded participants in *heritage culture maintenance* preference (central domain: *F* = 2.630, *p* = .105, η^2^ = .004; peripheral domain; *F* = 0.004, *p* = .950, η^2^ = .000), as well as in *destination culture adoption* preference (central domain: *F* = 2.403, *p* = .122, η^2^ = .003; peripheral domain; *F* = 0.026, *p* = .873, η^2^ = .000). Similarly, they did not significantly differ in their *contact quantity* (school: *F* = 1.248, *p* = .264, η^2^ = .002; out-of-school; *F* = 0.884, *p* = .348, η^2^ = .001), nor in levels of *negative contact* (school: *F* = 2.392, *p* = .122, η^2^ = .003; out-of-school; *F* = 0.078, *p* = .780, η^2^ = .000). However, participants in the final sample displayed slightly higher levels of *positive contact* in school (*F* = 5.153, *p* = .023, η^2^ = .007, Cohen’s *d* = .232) but not in the out-of-school context (*F* = 0.053, *p* = .819, η^2^ = .000).

At T2, adolescents in the final longitudinal sample did not significantly differ from the excluded participants in both *heritage culture maintenance* (central domain: *F* = 0.240, *p* = .624, η^2^ = .000; peripheral domain; *F* = 0.098, *p* = .754, η^2^ = .000) and *destination culture adoption* preference (central domain: *F* = 1.744, *p* = .187, η^2^ = .002; peripheral domain; *F* = 1.508, *p* = .220, η^2^ = .002), and they displayed similar levels of *positive contact* (school: *F* = 1.532, *p* = .216, η^2^ = .002; out-of-school; *F* = 1.118, *p* = .291, η^2^ = .001). However, adolescents from the final longitudinal sample reported slightly lower levels of *contact quantity* (*F* = 5.840, *p* = .016, η^2^ = .008, Cohen’s *d* = -.354) and *negative contact* (*F* = 5.376, *p* = .021, η^2^ = .007, Cohen’s *d* = -.339) in the school context, while no differences emerged in the out-of-school context (contact quantity: *F* = 3.485, *p* = .062, η^2^ = .005; negative contact; *F* = 3.596, *p* = .058, η^2^ = .005).

At T3, no significant differences emerged among the adolescents in the final longitudinal sample from the excluded ones in *heritage culture maintenance* preference (central domain: *F* = 2.593, *p* = .108, η^2^ = .004; peripheral domain; *F* = 0.015, *p* = .904, η^2^ = .000). However, they displayed lower *destination culture adoption* preference in central domains (*F* = 11.488, *p* < .001, η^2^ = .017, Cohen’s *d* = -.704), while no significant difference was found in peripheral domains (*F* = 0.143, *p* = .705, η^2^ = .000). Moreover, adolescents in the final longitudinal sample did not differ from the excluded ones in their *contact quantity* (school: *F* = 0.361, *p* = .548, η^2^ = .001; out-of-school; *F* = 2.469, *p* = .117, η^2^ = .004), along with *positive* (school: *F* = 1.046, *p* = .307, η^2^ = .002; out-of-school; *F* = 0.000, *p* = .987, η^2^ = .000) and *negative contact* (school: *F* = 1.355, *p* = .245, η^2^ = .002; out-of-school; *F* = 0.002, *p* = .968, η^2^ = .000).

## Ethnic Minority Group Comparisons

At T1, ethnic minority group adolescents in the final longitudinal sample did not significantly differ from the excluded participants in *cultural heritage maintenance* (central domain: *F* = 0.010, *p* = .921, η^2^ = .000; peripheral domain; *F* = 0.001, *p* = .972, η^2^ = .000), as well as in *destination culture adoption* preferences (central domain: *F* = 0.003, *p* = .956, η^2^ = .000; peripheral domain; *F* = 2.430, *p* = .120, η^2^ = .010). Similarly, participants in the final longitudinal sample did not significantly differ in their *quantity of contact* (school: *F* = 0.139, *p* = .710, η^2^ = .001; out-of-school; *F* = 0.526, *p* = .469, η^2^ = .002), *positive contact* (school: *F* = 0.011, *p* = .917, η^2^ = .000; out-of-school; *F* = 0.136, *p* = .713, η^2^ = .001), and *negative contact* (school: *F* = 0.627, *p* = .429, η^2^ = .003; out-of-school; *F* = 2.210, *p* = .138, η^2^ = .009).

At T2, adolescents in the final longitudinal sample did not differ from the excluded ones in *cultural heritage maintenance* (central domain: *F* = 0.578, *p* = .448, η^2^ = .003; peripheral domain; *F* = 0.116, *p* = .733, η^2^ = .001). While there were no significant differences in *destination culture adoption* in central domains (*F* = 1.291, *p* = .257, η^2^ = .006), adolescents in the final longitudinal sample displayed slightly higher *destination culture adoption* in peripheral domains (*F* = 5.558, *p* = .019, η^2^ = .025, Cohen’s *d* = .501), along with *contact quantity* (school: F = 21.764, *p* < .001, η^2^ = .090, Cohen’s *d* = .974; out-of-school; *F* = 11.818, *p* < .001, η^2^ = .051, Cohen’s *d* = .718), and *positive contact* (school: *F* = 6.015, *p* = .015, η^2^ = .027, Cohen’s *d* = .512; out-of-school; *F* = 3.985, *p* = .047, η^2^ = .018, Cohen’s *d* = .424). There were no significant differences in their *negative contact* (school: *F* = 2.939, *p* = .088, η^2^ = .013; out-of-school; *F* = 3.171, *p* = .076, η^2^ = .014).

At T3, adolescents in the final longitudinal sample did not significantly differ from the excluded participants in *cultural heritage maintenance* (central domain: *F* = 0.738, *p* = .391, η^2^ = .004; peripheral domain; *F* = 0.430, *p* = .513, η^2^ = .002), *destination culture adoption* preference (central domain: *F* = 0.377, *p* = .540, η^2^ = .002; peripheral domain; *F* = 0.000, *p* = .987, η^2^ = .000), as well as in *contact quantity* (school; *F* = 0.403, *p* = .526, η^2^ = .002; out-of-school; *F* = 1.966, *p* = .162, η^2^ = .010), *positive* (school: *F* = 1.811, *p* = .180, η^2^ = .009; out-of-school; *F* = 3.412, *p* = .066, η^2^ = .017) and *negative contact* (school: *F* = 1.122, *p* = .291, η^2^ = .006; out-of-school; *F* = 0.015, *p* = .902, η^2^ = .000).

# Attrition Within the Final Longitudinal Sample

A total of 984 adolescents were included in the final longitudinal sample. Of these, 740 were majority Italian adolescents, while the remaining 244 were ethnic minority adolescents. Adolescents included completed at least two out of the three waves of the current study. However, participation across the waves varied. Among the 740 majority participants, 562 (75.95%) completed all three assessments and 178 (24.05%) completed only two. Among the 244 ethnic minority adolescents, 148 (60.66%) completed all assessments and 96 (39.34%) participated only twice.

To further examine missingness within the final longitudinal sample, several attritions checks were performed. First, chi-square tests were used to examine possible differences on key demographics between adolescents who participated in all three assessments and those who completed only two. Results are presented in Table S1. Next, univariate ANOVAs were conducted to examine whether adolescents with varying participation rates differed in age and mean scores on the main study variables (i.e., acculturation preferences and intergroup contact) aggregated across time points. Results are displayed in Table S2. These analyses were conducted separately for majority and ethnic minority adolescents.

## Majority Group Comparisons

Attrition analyses revealed a few differences between adolescents who participated twice and those who participated three times in the study, although differences were of small magnitude. For what concerns demographics, male adolescents and older adolescents were more likely to complete only two out of the three assessments. For what concerns the study variables, those who completed only two assessments had slightly higher preference for the destination culture adoption in the central domain but no differences emerged in the other acculturation preferences. Additionally, adolescents who completed only two assessments reported slightly more frequent intergroup contact experiences and more negative contact in both the school and out-of-school contexts.

## Ethnic Minority Group Comparisons

Ethnic minority adolescents who participated in two assessments did not differ in terms of gender from those who participated in all three assessments, while a marginal difference emerged in terms of migrant generation with first-generation adolescents slightly more likely to complete questionnaires only twice. Additionally, older adolescents were more likely to complete only two out of the three questionnaires. For what concerns the study variables, ethnic minority adolescents who completed only two assessments had slightly higher heritage culture maintenance in both central and peripheral domains, and reported slightly less positive and more negative intergroup contact in the out-of-school context. Differences were overall of small magnitude.

## Table S1

*Results of Chi-square analyses*

|  | Adolescent participation in | | | | | |
| --- | --- | --- | --- | --- | --- | --- |
|  | 2 waves | 3 waves | χ^2^ | df | *p* | Cramer’s V |
| **Majority group comparisons** | | | | | | |
| **Adolescent sex** |  |  | 19.005 | 1 | < .001 | .160 |
| Male | **87 (+)** | 174 |  |  |  |  |
| Female | **91 (-)** | 388 |  |  |  |  |
| **Ethnic Minority group comparisons** | | | | | | |
| **Adolescent sex** |  |  | 1.960 | 1 | .162 | .090 |
| Male | 47 | 59 |  |  |  |  |
| Female | 49 | 89 |  |  |  |  |
| **Immigrant generation** |  |  | 3.955 | 1 | .047 | .127 |
| First-generation | **31 (+)** | **31 (-)** |  |  |  |  |
| Second-generation | 65 | 117 |  |  |  |  |

*Note.* Counts are reported in cells. Bold values indicate that the observed count is significantly different from the expected count based on standardized residuals: (+) indicates that the observed value is higher than expected, while (-) indicates that the observed value is lower than expected. Cramer’s V is reported as an effect size for Chi-square tests (values ≈ .10 indicate a small effect, ≈ .30 a medium effect, and ≈ .50 a large effect; Cohen, 1988).

## Table S2

*Results of univariate ANOVAs*

|  | Adolescent participation in | | | | | |
| --- | --- | --- | --- | --- | --- | --- |
|  | 2 waves | 3 waves | *F* | df | *p* | η^2^ |
| **Majority group comparisons** | | | | | | |
| **Adolescent age at T1** | 14.89 (0.79) | 14.49 (0.61) | 50.409 | 1 | < .001 | .064 |
| **Maintenance - central** | 3.42 (0.75) | 3.50 (0.72) | 1.705 | 1 | .192 | .002 |
| **Adoption - central** | 2.82 (0.84) | 2.62 (0.77) | 9.149 | 1 | .003 | .012 |
| **Maintenance – peripheral** | 2.91 (0.64) | 2.92 (0.59) | 0.081 | 1 | .776 | .000 |
| **Adoption - peripheral** | 3.20 (0.63) | 3.13 (0.58) | 2.280 | 1 | .131 | .003 |
| **Quantity of contact – school** | 3.82 (0.97) | 3.60 (0.86) | 8.587 | 1 | .003 | .012 |
| **Positive contact – school** | 4.01 (0.73) | 4.03 (0.66) | 0.111 | 1 | .740 | .000 |
| **Negative contact – school** | 1.70 (0.65) | 1.59 (0.54) | 5.102 | 1 | .024 | .007 |
| **Quantity of contact – out-of-school** | 3.51 (1.09) | 3.15 (0.94) | 17.313 | 1 | < .001 | .023 |
| **Positive contact – out-of-school** | 3.76 (0.87) | 3.72 (0.81) | 0.482 | 1 | .488 | .001 |
| **Negative contact – out-of-school** | 1.78 (0.68) | 1.65 (0.63) | 5.809 | 1 | .016 | .008 |
| **Ethnic Minority group comparisons** | | | | | | |
| **Adolescent age at T1** | 15.12 (0.92) | 14.77 (0.76) | 10.767 | 1 | .001 | .043 |
| **Maintenance - central** | 3.12 (0.95) | 2.76 (0.95) | 8.100 | 1 | .005 | .033 |
| **Adoption - central** | 3.07 (0.94) | 3.20 (0.88) | 1.213 | 1 | .272 | .005 |
| **Maintenance – peripheral** | 2.94 (0.95) | 2.51 (1.03) | 9.697 | 1 | .001 | .039 |
| **Adoption - peripheral** | 3.68 (0.75) | 3.84 (0.77) | 2.222 | 1 | .137 | .009 |
| **Quantity of contact – school** | 4.59 (0.70) | 4.73 (0.48) | 3.535 | 1 | .061 | .015 |
| **Positive contact – school** | 4.13 (0.64) | 4.27 (0.63) | 2.888 | 1 | .091 | .012 |
| **Negative contact – school** | 1.79 (0.62) | 1.76 (0.64) | 0.089 | 1 | .766 | .000 |
| **Quantity of contact – out-of-school** | 4.42 (0.78) | 4.54 (0.71) | 1.513 | 1 | .220 | .006 |
| **Positive contact – out-of-school** | 4.12 (0.70) | 4.35 (0.60) | 7.392 | 1 | .007 | .030 |
| **Negative contact – out-of-school** | 1.81 (0.70) | 1.63 (0.55) | 5.189 | 1 | .024 | .021 |

*Note.* Means and standard deviations (in parenthesis) are reported in the cells.

# Descriptives and Correlations among Study Variables

## Table S3

*Means (M), Standard Deviations (SDs), Cronbach's Alpha (α) and Intraclass Correlation Coefficients (ICC) of Study Variables*

|  | **ICC** | **T1** | | |  | **T2** | | |  | **T3** | | | | |
| --- | --- | --- | --- | --- | --- | --- | --- | --- | --- | --- | --- | --- | --- | --- |
|  |  | *M* | *SD* | *α* |  | *M* | *SD* | *α* |  | *M* | | *SD* | *α* |  |
| **Majority group** | | | | | | | | | | | | | | |
| Maintenance domains |  |  |  |  |  |  |  |  |  |  |  | |  |  |
| Central | .410 | 3.560 | 0.948 | .760 |  | 3.433 | 0.922 | .766 |  | 3.473 | 0.865 | | .825 |  |
| Peripheral | .353 | 2.945 | 0.841 | .700 |  | 2.879 | 0.799 | .689 |  | 2.940 | 0.709 | | .664 |  |
| Adoption domains |  |  |  |  |  |  |  |  |  |  |  | |  |  |
| Central | .417 | 2.672 | 1.059 | .817 |  | 2.677 | 0.982 | .807 |  | 2.602 | 0.907 | | .838 |  |
| Peripheral | .344 | 3.209 | 0.818 | .709 |  | 3.188 | 0.767 | .688 |  | 3.018 | 0.693 | | .669 |  |
| Contact in school |  |  |  |  |  |  |  |  |  |  |  | |  |  |
| Quantity | .390 | 3.916 | 1.029 |  |  | 3.594 | 1.158 |  |  | 3.423 | 1.124 | |  |  |
| Positive | .504 | 4.061 | 0.773 | .898 |  | 3.989 | 0.851 | .942 |  | 4.020 | 0.808 | | .945 |  |
| Negative | .387 | 1.582 | 0.694 | .840 |  | 1.608 | 0.720 | .888 |  | 1.651 | 0.737 | | .897 |  |
| Contact out-of-school |  |  |  |  |  |  |  |  |  |  |  | |  |  |
| Quantity | .467 | 3.197 | 1.213 |  |  | 3.267 | 1.215 |  |  | 3.177 | 1.167 | |  |  |
| Positive | .452 | 3.667 | 1.050 | .954 |  | 3.754 | 0.966 | .963 |  | 3.762 | 0.977 | | .962 |  |
| Negative | .416 | 1.628 | 0.777 | .907 |  | 1.659 | 0.779 | .917 |  | 1.732 | 0.831 | | .922 |  |
| **Ethnic minority group** | | | | | | | | | | | | | | |
| Maintenance domains |  |  |  |  |  |  |  |  |  |  |  | |  |  |
| Central | .501 | 2.833 | 1.099 | .692 |  | 2.977 | 1.136 | .769 |  | 2.764 | 1.080 | | .790 |  |
| Peripheral | .410 | 2.697 | 1.232 | .884 |  | 2.653 | 1.255 | .868 |  | 2.489 | 1.157 | | .888 |  |
| Adoption domains |  |  |  |  |  |  |  |  |  |  |  | |  |  |
| Central | .427 | 3.126 | 1.054 | .727 |  | 3.133 | 1.022 | .723 |  | 3.110 | 1.057 | | .821 |  |
| Peripheral | .346 | 3.830 | 0.854 | .799 |  | 3.826 | 0.881 | .820 |  | 3.662 | 0.994 | | .898 |  |
| Contact in school |  |  |  |  |  |  |  |  |  |  |  | |  |  |
| Quantity | .208 | 4.735 | 0.675 |  |  | 4.687 | 0.658 |  |  | 4.624 | 0.832 | |  |  |
| Positive | .426 | 4.178 | 0.736 | .919 |  | 4.225 | 0.706 | .935 |  | 4.185 | 0.799 | | .951 |  |
| Negative | .343 | 1.732 | 0.743 | .866 |  | 1.800 | 0.824 | .915 |  | 1.826 | 0.797 | | .891 |  |
| Contact out-of-school |  |  |  |  |  |  |  |  |  |  |  | |  |  |
| Quantity | .376 | 4.544 | 0.779 |  |  | 4.526 | 0.809 |  |  | 4.456 | 0.946 | |  |  |
| Positive | .426 | 4.257 | 0.768 | .950 |  | 4.241 | 0.705 | .942 |  | 4.260 | 0.793 | | .950 |  |
| Negative | .233 | 1.560 | 0.696 | .886 |  | 1.709 | 0.813 | .932 |  | 1.767 | 0.806 | | .910 |  |

## Table S4

*Correlations among Study Variables for Italian Majority (above the diagonal) and Ethnic Minority (below the diagonal) Participants*

|  | 1. | 2. | 3. | 4. | 5. | 6. | 7. | 8. | 9. | 10. | 11. | 12. | 13. | 14. | 15. | 16. | 17. |
| --- | --- | --- | --- | --- | --- | --- | --- | --- | --- | --- | --- | --- | --- | --- | --- | --- | --- |
| 1.Age |  | -.11^**^ | -.02 | -.06 | .03 | .06 | .04 | -.01 | .05 | .00 | -.05 | .02 | .01 | -.06 | -.01 | .04 | -.01 |
| 2.Gender | -.04 |  | -.02 | .12^**^ | -.18^***^ | -.19^***^ | -.01 | .15^***^ | -.20^***^ | -.04 | .10^*^ | -.22^***^ | .00 | .04 | -.10^**^ | -.13^***^ | -.05 |
| 3.Maint. Per. T1 | -.05 | -.06 |  | .46^***^ | -.03 | -.01 | .04 | .12^**^ | -.08 | .11^**^ | .12^**^ | -.09^*^ | .34^***^ | .27^***^ | -.12^**^ | -.10^*^ | .05 |
| 4.Maint. Cen. T1 | -.01 | -.01 | .66^***^ |  | -.12^**^ | -.29^***^ | .03 | .23^***^ | -.19^***^ | .15^***^ | .23^***^ | -.14^***^ | .27^***^ | .41^***^ | -.16^***^ | -.31^***^ | .05 |
| 5.Adop. Per. T1 | .05 | .03 | -.03 | -.12 |  | .65^***^ | .02 | -.07 | .16^***^ | -.02 | -.14^***^ | .19^***^ | -.05 | -.16^***^ | .43^***^ | .36^***^ | -.09^*^ |
| 6.Adop. Cen. T1 | -.02 | -.01 | -.02 | -.04 | .61^***^ |  | .04 | -.17^***^ | .27^***^ | -.01 | -.14^***^ | .23^***^ | -.01 | -.26^***^ | .38^***^ | .48^***^ | -.03 |
| 7.Cont. Sch. T1 | -.20^***^ | .01 | -.05 | -.02 | .39^***^ | .10 |  | .40^***^ | -.11^**^ | .30^***^ | .23^***^ | -.04 | .05 | .09^*^ | -.02 | -.02 | .36^***^ |
| 8.Pos. C. Sch. T1 | -.04 | -.06 | -.10 | -.20^***^ | .33^***^ | .18^*^ | .41^***^ |  | -.54^***^ | .21^***^ | .47^***^ | -.32^***^ | .17^***^ | .22^***^ | -.14^***^ | -.18^***^ | .21^***^ |
| 9.Neg. C. Sch. T1 | .05 | -.02 | .13 | .13 | -.22^**^ | -.12 | -.10 | -.61^***^ |  | -.03 | -.30^***^ | .62^***^ | -.08^*^ | -.19^***^ | .23^***^ | .25^***^ | -.05 |
| 10. Cont. Out T1 | -.22^***^ | .00 | -.03 | -.04 | .33^***^ | .19^*^ | .71^***^ | .45^***^ | -.13 |  | .56^***^ | -.01 | .05 | .08^*^ | -.04 | -.10^*^ | .27^***^ |
| 11.Pos. C. Out T1 | -.03 | -.-6 | -.04 | -.11 | .31^***^ | .21^**^ | .43^***^ | .70^***^ | -.34^***^ | .63^***^ |  | -.37^***^ | .09^*^ | .18^***^ | -.07 | -.14^***^ | .19^***^ |
| 12.Neg. C. Out T1 | .04 | .01 | .11 | .06 | -.09 | -.01 | -.09 | -.33^***^ | .58^***^ | -.09 | -.38^***^ |  | -.07 | -.14^***^ | .19^***^ | .20^***^ | -.07 |
| 13.Maint. Per. T2 | .07 | -.09 | .45^***^ | .33^***^ | -.09 | .01 | -.13 | -.09 | .12 | -.05 | .08 | .07 |  | .49^***^ | -.10^*^ | -.01 | .12^**^ |
| 14.Maint. Cen. T2 | .04 | -.05 | .42^***^ | .60^***^ | -.14 | .00 | -.12 | -.21^*^ | .18^*^ | -.06 | -.07 | .08 | .70^***^ |  | -.13^***^ | -.32^***^ | .14^***^ |
| 15.Adop. Per. T2 | -.07 | .08 | -.10 | -.10 | ..40^***^ | .33^***^ | .20^*^ | .27^**^ | -.23^**^ | .28^***^ | .21^*^ | -.21^*^ | .02 | -.03 |  | .58^***^ | -.01 |
| 16.Adop. Cen. T2 | -.03 | -.02 | -.01 | .05 | .25^**^ | .46^***^ | .12 | .20^*^ | -.09 | .21^*^ | .18^*^ | -.05 | .08 | .00 | .60^***^ |  | -.05 |
| 17. Cont. Sch. T2 | -.13 | .13 | -.11 | -.05 | .07 | .04 | .18^*^ | .14 | -.25^**^ | .14 | .07 | -.11 | -.04 | -.05 | .25^***^ | .10 |  |
| 18.Pos. C. Sch. T2 | -.06 | .03 | -.07 | -.13 | .27^***^ | .14 | .15 | .59^***^ | -.55^***^ | .25^**^ | .47^***^ | -.29^***^ | .07 | .00 | .37^***^ | .24^***^ | .44^***^ |
| 19.Neg. C. Sch.T2 | .09 | -.13 | .11 | .12 | -.11 | -.07 | -.13 | -.42^***^ | .56^***^ | -.07 | -.23^**^ | .32^***^ | .08 | .08 | -.10 | .07 | -.20^**^ |
| 20. Cont. Out T2 | -.11 | .09 | -.14 | -.13 | .16^*^ | .10 | .11 | .25^**^ | -.24^**^ | .36^***^ | .28^***^ | -.09 | -.07 | -.09 | .25^***^ | .11 | .46^***^ |
| 21.Pos. C. Out T2 | -.08 | .02 | -.08 | -.09 | .28^***^ | .20^**^ | .17^*^ | .53^***^ | -.50^***^ | .35^***^ | .55^***^ | -.51^***^ | -.03 | -.02 | .42^***^ | .32^***^ | .35^***^ |
| 22.Neg. C. Out T2 | .14^*^ | -.09 | .13 | .12 | -.13 | -.11 | -.16 | -.34^***^ | .47^***^ | -.09 | -.28^***^ | .44^***^ | .10 | .07 | -.07 | .07 | -.25^***^ |
| 23.Maint. Per. T3 | -.01 | -.11 | .38^***^ | .20^***^ | .02 | .06 | -.10 | -.01 | .04 | .02 | .06 | .07 | .43^***^ | .28^***^ | -.09 | -.04 | -.12 |
| 24.Maint. Cen. T3 | -.01 | -.01 | .27^***^ | .40^***^ | .01 | .01 | -.02 | -.07 | .16 | .05 | .07 | .12 | .36^***^ | .51^***^ | -.08 | -.07 | -.04 |
| 25.Adop. Per. T3 | -.07 | .11 | .09 | -.04 | .39^***^ | .44^***^ | .24^**^ | .22^*^ | -.12 | .29^**^ | .32^***^ | -.07 | .13 | .10 | .26^***^ | .20^*^ | .19^*^ |
| 26.Adop. Cen. T3 | -.09 | .07 | .16 | .11 | .28^**^ | .46^***^ | .22^*^ | .18^*^ | -.09 | .26^**^ | .31^***^ | -.10 | .25^**^ | .19^*^ | .09 | .31^***^ | .06 |
| 27. Cont. Sch. T3 | -.09 | .13 | .02 | .03 | .17 | .13 | .19^*^ | .15 | -.12 | .15 | .13 | .00 | .10 | .07 | .18^*^ | -.01 | .27^***^ |
| 28.Pos. C. Sch. T3 | -.11 | .17^*^ | .07 | .00 | .28^**^ | .25^**^ | .19^*^ | .46^***^ | -.40^***^ | .22^*^ | .30^***^ | -.28^**^ | .16^*^ | .15 | .28^***^ | .11 | .19^*^ |
| 29.Neg. C. Sch.T3 | -.05 | -.17^*^ | .07 | .06 | -.22^*^ | -.10 | -.14 | -.36^***^ | .37^***^ | -.13 | -.18^*^ | .27^**^ | -.09 | -.07 | -.10 | -.07 | -.16 |
| 30.Contact Out T3 | -.01 | .17^*^ | -.06 | -.11 | .32^***^ | .21^*^ | .23^*^ | .36^***^ | -.20^*^ | .34^***^ | .36^***^ | .00 | .09 | .00 | .22^**^ | .15 | .35^***^ |
| 31.Pos. C. Out T3 | -.13 | .13 | .04 | .01 | .23^**^ | .20^*^ | .21^*^ | .41^***^ | -.23^**^ | .27^**^ | .40^***^ | -.19^*^ | .17^*^ | .09 | .16^*^ | .09 | .20^*^ |
| 32.Neg. C. Out T3 | .09 | -.07 | -.08 | -.07 | -.03 | -.03 | -.02 | -.30^***^ | .29^***^ | -.06 | -.21^*^ | .25^**^ | -.12 | -.14 | -.17^*^ | -.04 | .16^*^ |

**Table S4 (continued)**

*Correlations among Study Variables for Italian Majority (above the diagonal) and Ethnic Minority (below the diagonal) Participants*

|  | 18. | 19. | 20. | 21. | 22. | 23. | 24. | 25. | 26. | 27. | 28. | 29. | 30. | 31. | 32. |
| --- | --- | --- | --- | --- | --- | --- | --- | --- | --- | --- | --- | --- | --- | --- | --- |
| 1.Age | -.04 | -.02 | .05 | -.03 | .03 | .00 | -.12^**^ | .03 | .08^*^ | -.02 | -.08^*^ | .11^**^ | .06 | -.06 | .08^*^ |
| 2.Gender | .15^***^ | -.24^***^ | -.11^**^ | .09^*^ | -.21^***^ | -.04 | .07 | -.04 | -.14^***^ | .02 | .19^***^ | -.22^***^ | .03 | .16^***^ | -.20^***^ |
| 3.Maint. Per. T1 | .15^***^ | -.12^**^ | .05 | .11^**^ | -.11^**^ | .34^***^ | .22^***^ | -.03 | .01 | .06 | .17^***^ | -.08 | .02 | .09^*^ | -.08 |
| 4.Maint. Cen. T1 | .23^***^ | -.21^***^ | .12^**^ | .18^***^ | -.16^***^ | .26^***^ | .38^***^ | -.04 | -.13^**^ | .07 | .21^***^ | -.17^***^ | .05 | .21^***^ | -.14^**^ |
| 5.Adop. Per. T1 | -.19^***^ | .15^***^ | -.02 | -.11^**^ | .10^*^ | -.11^**^ | -.14^**^ | .31^***^ | .31^***^ | -.06 | -.22^***^ | .16^***^ | .02 | -.10^*^ | .15^***^ |
| 6.Adop. Cen. T1 | -.21^***^ | .16^***^ | -.02 | -.14^***^ | .10^*^ | -.09^*^ | -.25^***^ | .27^***^ | .39^***^ | -.02 | -.24^***^ | .19^***^ | .02 | -.13^**^ | .18^***^ |
| 7.Cont. Sch. T1 | .26^***^ | -.14^***^ | .22^***^ | .20^***^ | -.09^*^ | .01 | .02 | .02 | -.06 | .37^***^ | .19^***^ | -.06 | .19^***^ | .14^***^ | -.05 |
| 8.Pos. C. Sch. T1 | .53^***^ | -.38^***^ | .18^***^ | .42^***^ | -.31^***^ | .15^***^ | .13^**^ | -.06 | -.11^*^ | .20^***^ | .44^***^ | -.26^***^ | .10^*^ | .32^***^ | -.27^***^ |
| 9.Neg. C. Sch. T1 | -.36^***^ | .47^***^ | .02 | -.25^***^ | .41^***^ | -.11^*^ | -.13^**^ | .12^**^ | .17^***^ | -.05 | -.29^***^ | .30^***^ | .04 | -.18^***^ | .32^***^ |
| 10. Cont. Out T1 | .22^***^ | -.02 | .46^***^ | .29^***^ | -.01 | .03 | .05 | -.01 | .00 | .33^***^ | .23^***^ | -.01 | .43^***^ | .30^***^ | .01 |
| 11.Pos. C. Out T1 | .42^***^ | -.25^***^ | .34^***^ | .48^***^ | -.24^***^ | .04 | .11^**^ | -.07 | -.11^**^ | .23^***^ | .39^***^ | -.18^***^ | .20^***^ | .39^***^ | -.20^***^ |
| 12.Neg. C. Out T1 | -.30^***^ | .43^***^ | .00 | -.25^***^ | .41^***^ | -.07 | -.14^***^ | .11^**^ | .18^***^ | -.03 | -.27^***^ | .32^***^ | .06 | -.22^***^ | .39^***^ |
| 13.Maint. Per. T2 | .16^***^ | -.14^***^ | .07 | .12^**^ | -.08^*^ | .41^***^ | .22^***^ | -.02 | .03 | .07 | .18^***^ | -.07 | .03 | .10^*^ | -.07 |
| 14.Maint. Cen. T2 | .24^***^ | -.15^***^ | .08^*^ | .21^***^ | -.09^*^ | .30^***^ | .47^***^ | -.04 | -.21^***^ | .06 | .21^***^ | -.14^***^ | .04 | .16^***^ | -.10^*^ |
| 15.Adop. Per. T2 | -.13^***^ | .17^***^ | .01 | -.10^**^ | .12^**^ | -.15^***^ | -.16^***^ | .32^***^ | .27^***^ | -.01 | -.14^***^ | .16^***^ | .05 | -.08^*^ | .10^**^ |
| 16.Adop. Cen. T2 | -.18^***^ | .19^***^ | -.01 | -.16^***^ | .13^***^ | -.11^**^ | -.30^***^ | .26^***^ | .37^***^ | -.05 | -.20^***^ | .14^***^ | .00 | -.19^***^ | .13^**^ |
| 17. Cont. Sch. T2 | .47^***^ | -.06 | .39^***^ | .27^***^ | -.11^**^ | .01 | .07 | -.02 | -.05 | .50^***^ | .30^***^ | -.01 | .30^***^ | .20^***^ | -.03 |
| 18.Pos. C. Sch. T2 |  | -.48^***^ | .28^***^ | .56^***^ | -.41^***^ | .11^**^ | .15^***^ | -.11^**^ | -.21^***^ | .27^***^ | .55^***^ | -.30^***^ | .20^***^ | .37^***^ | -.26^***^ |
| 19.Neg. C. Sch.T2 | -.46^***^ |  | .00 | -.30^***^ | .74^***^ | -.13^**^ | -.15^***^ | .07 | .14^***^ | -.05 | -.28^***^ | .41^***^ | -.02 | -.23^***^ | .39^***^ |
| 20. Cont. Out T2 | .20^**^ | -.13 |  | .55^***^ | -.01 | .04 | .01 | .04 | .05 | .33^***^ | .16^***^ | .05 | .48^***^ | .30^***^ | .05 |
| 21.Pos. C. Out T2 | .72^***^ | -.37^***^ | .37^***^ |  | -.42^***^ | .12^**^ | .14^***^ | -.02 | -.07 | .20^***^ | .34^***^ | -.19^***^ | .22^***^ | .46^***^ | -.24^***^ |
| 22.Neg. C. Out T2 | -.34^***^ | .78^***^ | -.21^**^ | -.40^***^ |  | -.13^***^ | -.15^***^ | .04 | .07 | -.06 | -.27^***^ | .38^***^ | -.01 | -.28^***^ | .47^***^ |
| 23.Maint. Per. T3 | -.05 | .00 | .03 | -.04 | .04 |  | .50^***^ | .01 | .10^*^ | .04 | .14^***^ | -.09^*^ | .08^*^ | .15^***^ | -.07 |
| 24.Maint. Cen. T3 | -.04 | .00 | .05 | -.01 | -.01 | .74^***^ |  | .00 | -.22^***^ | .04 | .20^***^ | -.19^***^ | .05 | .19^***^ | -.14^***^ |
| 25.Adop. Per. T3 | .19^*^ | -.15 | .22^**^ | .22^**^ | -.19^*^ | .11 | .18^*^ |  | .56^***^ | .05 | -.12^**^ | .11^**^ | .08^*^ | -.05 | .13^***^ |
| 26.Adop. Cen. T3 | .14 | -.05 | .08 | .17^*^ | -.10 | .23^**^ | .24^***^ | .67^***^ |  | .00 | -.18^***^ | .24^***^ | .05 | -.06 | .20^***^ |
| 27. Cont. Sch. T3 | .08 | -.03 | .24^**^ | .17^*^ | .01 | .01 | .00 | .17^*^ | .06 |  | .43^***^ | -.07 | .48^***^ | .31^***^ | -.10^**^ |
| 28.Pos. C. Sch. T3 | .35^***^ | -.30^***^ | .23^**^ | .38^***^ | -.19^*^ | .01 | .02 | .29^***^ | .19^**^ | .58^***^ |  | -.44^***^ | .23^***^ | .51^***^ | -.38^***^ |
| 29.Neg. C. Sch.T3 | -.24^**^ | .26^***^ | -.17^*^ | -.25^**^ | .18^*^ | .09 | .13 | -.22^**^ | -.11 | -.33^***^ | -.66^***^ |  | -.04 | -.29^***^ | .70^***^ |
| 30.Contact Out T3 | .25^**^ | -.13 | .40^***^ | .31^***^ | -.07 | .01 | .02 | .27^***^ | .13 | .60^***^ | .52^***^ | -.44^***^ |  | .49^***^ | -.01 |
| 31.Pos. C. Out T3 | .34^***^ | -.19^*^ | .24^**^ | .34^***^ | -.16^*^ | .000 | .02 | .32^***^ | .26^***^ | .47^***^ | .70^***^ | -.55^***^ | .58^***^ |  | -.38^***^ |
| 32.Neg. C. Out T3 | -.21^*^ | .11 | -.10 | -.24^***^ | .09 | .06 | .08 | -.19^**^ | -.12 | -.42^***^ | -.59^***^ | .65^***^ | -.36^***^ | -.56^***^ |  |

*Note.* Maint.=heritage culture maintenance; Per.=peripheral domain; Cen.=central domain ; Adop.=destination culture adoption; Cont.=quantity of contact; Sch.=school context; Pos.=positive contact; Neg.=negative contact; Out=out-of-school context; T = Time. ^*^ *p* < .05; ^**^ *p* < .01, ^***^ *p* < .001

# Confirmatory Factor Analysis

As a preliminary step, a set of confirmatory factor analyses (CFAs) were conducted in M*plus* to examine the factorial structure of the *acculturation preferences across peripheral and central domains*. To this end, acculturation preferences at T1 were used. These analyses were conducted separately for majority and ethnic minority adolescents, and separately for *heritage culture maintenance* and *destination culture adoption* scales. This model reduced complexity and allowed for a direct examination of each scale’s factorial structure without additional noise (i.e., covariance between maintenance and adoption scores). First, a one-factor model (i.e., 6 items loading on a single latent indicator) was tested (M1). Next, a two-factor model was tested, which included two latent indicators (e.g., one capturing *acculturation preference in central domains,* and the other capturing *acculturation preference in peripheral domains*), each defined by three items (M2). The two latent factors were allowed to correlate in the model.

The fit of each model was examined based on a combination of indices: the Comparative Fit Index (CFI) with values higher than .90 and .95 indicative of an acceptable and excellent fit, respectively; the Root Mean Square Error of Approximation (RMSEA) and the Standardized Root Mean Square Residual (SRMR) with values lower than .08 and .05 indicative of acceptable and excellent fit, respectively; and the RMSEA 90% confidence interval’s upper bound lower than .10 (Chen et al., 2008). The best fitting and most parsimonious model was retained. Results are reported in Table S5. As can be inferred, the two-factors models represented acculturation preferences across domains better for both ethnic majority and minority participants and was therefore retained for subsequent analyses.

## Table S5

*Confirmatory Factor Analysis: Model Fit*

|  | **Model fit indices** | | | | | | | | | | | |
| --- | --- | --- | --- | --- | --- | --- | --- | --- | --- | --- | --- | --- |
|  | | **Majority group** | | | | |  | **Minority group** | | | | |
|  | | *χ*_SB_^2^ | *df* | CFI | SRMR | RMSEA  [90% CI] |  | *χ*_SB_^2^ | *df* | CFI | SRMR | RMSEA  [90% CI] |
| **Maintenance domains** | | | | | | | | | | | | |
| M1. One factor model | | 151.661 | 9 | .804 | .076 | .157  [.136, .180] |  | 12.557 | 9 | .989 | .036 | .048  [.000, .106] |
| M2. Two factors model | | 67.693 | 8 | .918 | .048 | .108  [.085, .132] |  | 5.907 | 8 | 1.000 | .022 | .000  [.000, .073] |
| **Adoption domains** | | | | | | | | | | | | |
| M1. One factor model | | 49.176 | 9 | .960 | .035 | .084  [.062, .107] |  | 25.365 | 9 | .934 | .046 | .104  [.058, .153] |
| M2. Two factors model | | 24.078 | 8 | .984 | .024 | .056  [.031, .083] |  | 9.465 | 8 | .994 | .028 | .033  [.000, .100] |

*Note*. M = Model; χ^2^ = Chi-square; df = Degree of Freedom; CFI = Comparative Fit Index; SRMR = Standardized Root Mean Square Residual;

RMSEA = Root Mean Square Error of Approximation; CI = Confidence Interval.

# Longitudinal Measurement Invariance Tests

The longitudinal measurement invariance of acculturation (separately for maintenance and adoption) and intergroup contact (positive and negative separately for in and out-of-school domains) scales was tested separately for the majority and ethnic minority groups. The configural models (M1s) serve as the baseline for testing measurement invariance and are thus expected to demonstrate a good fit, which is assessed according to the following criteria. The Comparative Fit Index (CFI) with values higher than .90 and .95 indicates an acceptable and excellent fit, respectively. The Root Mean Square Error of Approximation (RMSEA) and the Standardized Root Mean Residual (SRMR) with values below .08 and .05 are indicative of an acceptable and very good fit (Byrne, 2012). Additionally, the RMSEA’s 90% confidence interval’s upper bound lower than .10 indicates an acceptable model fit (Chen et al., 2008).

The metric models (M2s) assess the extent to which the scale items load on the latent construct in an equivalent manner across the time points. Thus, metric invariance ensures that respondents attribute the same meaning to the latent construct over time (van de Schoot et al., 2012). Last, scalar models (M3s) build upon the previous metric ones and examine whether the items’ intercepts are equivalent across time points, allowing for meaningful comparisons of scores over time (van de Schoot et al., 2012). Increasingly constrained models are compared against less constrained ones, namely M2s with M1s, and M3s with M2s, and changes in fit indices are evaluated (Cheung & Rensvold, 2002). Specifically, a significant Δχ_SB_^2^ (Satorra & Bentler, 2001), and ΔCFI ≥ -.010 supplemented by ΔRMSEA ≥ .015 (Chen et al., 2008) are indicative of non-invariance.

If full metric and/or scalar invariance requirements are not met, partially invariant models are tested. Specifically, partial metric invariant models (M2a) would constrain only some but not all items’ factors loadings, freeing the one that are significantly different across times. Ina similar way, partial scalar invariant models (M3a) would constrain only some items’ intercepts to equality leaving the others free to vary across time. To identify the factors loading(s) and/or intercept(s) that are not equivalent across time points, the Wald test is used with a significant score at *p* < .050 indicating a statistically significant difference between two parameters.

Results of longitudinal measurement invariance testing are displayed in Table S6. As can be inferred, all acculturation and contact quality scales showed full scalar invariance for the majority group. For the ethnic minority group, partial scalar invariance was reached for destination culture adoption in peripheral and central domains and for the negative out-of-school contact scales, while the remaining scales showed full scalar invariance. Overall, these results confirm the robustness of our measures and allow for testing RI-CLPMs to address the main research question.

## Table S6

*Longitudinal Measurement Invariance Tests for Each Study Measure*

|  | **Model fit indices** | | | | |  | **Model comparison** | | | |
| --- | --- | --- | --- | --- | --- | --- | --- | --- | --- | --- |
|  | *χ*_SB_^2^ | *df* | CFI | SRMR | RMSEA  [90% CI] |  | Models | Δχ_SB_^2^ | ΔCFI | ΔRMSEA |
| **Majority Group** | | | | | | | | | | |
| Maintenance domains | | | | | | | | | | |
| M1. Configural model | 405.648 | 102 | .913 | .053 | .064 [.057, .070] |  |  |  |  |  |
| M2. Metric model | 422.695 | 110 | .910 | .056 | .062 [.056, .068] |  | M2-M1 | 16.086 (8)^*^ | -.003 | -.002 |
| M3. Scalar model | 459.810 | 122 | .903 | .057 | .061 [.055, .067] |  | M3-M2 | 35.860 (12)^***^ | -.007 | -.001 |
| Adoption domains | | | | | | | | | | |
| M1. Configural model | 253.637 | 102 | .961 | .036 | .045 [.038, .052] |  |  |  |  |  |
| M2. Metric model | 262.034 | 110 | .961 | .037 | .043 [.037, .050] |  | M2-M1 | 6.090 (8) | .000 | -.002 |
| M3. Scalar model | 309.333 | 122 | .952 | .043 | .046 [.039, .052] |  | M3-M2 | 50.433 (12)^***^ | -.009 | .003 |
| PC in school | | | | | | | | | | |
| M1. Configural model | 135.115 | 72 | .989 | .026 | .034 [.025, .043] |  |  |  |  |  |
| M2. Metric model | 143.482 | 80 | .988 | .031 | .033 [.024, .041] |  | M2-M1 | 5.987 (8) | -.001 | -.001 |
| M3. Scalar model | 158.139 | 90 | .988 | .034 | .032 [.024, .040] |  | M3-M2 | 13.408 (10) | .000 | -.001 |
| PC out-of-school | | | | | | | | | | |
| M1. Configural model | 130.455 | 72 | .992 | .019 | .033 [.024, .042] |  |  |  |  |  |
| M2. Metric model | 142.116 | 80 | .992 | .023 | .032 [.024, .041] |  | M2-M1 | 9.449 (8) | .000 | -.001 |
| M3. Scalar model | 160.975 | 90 | .990 | .025 | .033 [.024, .041] |  | M3-M2 | 19.119 (10)^*^ | -.002 | .001 |
| NC in school | | | | | | | | | | |
| M1. Configural model | 143.124 | 72 | .977 | .028 | .037 [.028, .045] |  |  |  |  |  |
| M2. Metric model | 149.124 | 80 | .978 | .033 | .034 [.026, .043] |  | M2-M1 | 8.045 (8) | .001 | -.003 |
| M3. Scalar model | 16.677 | 90 | .975 | .036 | .034 [.026, .042] |  | M3-M2 | 18.517 (10)^*^ | -.003 | .000 |
| NC out-of-school | | | | | | | | | | |
| M1. Configural model | 143.427 | 72 | .983 | .028 | .037 [.028, .045] |  |  |  |  |  |
| M2. Metric model | 146.856 | 80 | .984 | .031 | .034 [.025, .042] |  | M2-M1 | 5.609 (8) | .001 | -.003 |
| M3. Scalar model | 169.242 | 90 | .981 | .037 | .035 [.026, .043] |  | M3-M2 | 24.273 (10)^**^ | -.003 | .001 |
| **Ethnic Minority Group** | | | | | | | | | | |
| Maintenance domains |  |  |  |  |  |  |  |  |  |  |
| M1. Configural model | 138.676 | 102 | .979 | .049 | .039 [.020, .054] |  |  |  |  |  |
| M2. Metric model | 155.521 | 110 | .974 | .054 | .042 [.025, .056] |  | M2-M1 | 18.615 (8)^*^ | -.005 | .003 |
| M3. Scalar model | 184.101 | 122 | .965 | .061 | .046 [.032, .059] |  | M3-M2 | 29.655 (12)^**^ | -.009 | .004 |
| Adoption domains |  |  |  |  |  |  |  |  |  |  |
| M1. Configural model | 134.492 | 102 | .978 | .062 | .037 [.016, .052] |  |  |  |  |  |
| M2. Metric model | 142.355 | 110 | .978 | .065 | .035 [.015, .051] |  | M2-M1 | 7.607 (8) | .000 | -.002 |
| M3. Scalar model | 170.662 | 122 | .966 | .069 | .041 [.025, .055] |  | M3-M2 | 29.168 (12)^**^ | -.012 | .006 |
| M3a. Partial scalar model^1^ | 169.091 | 118 | .970 | .068 | .039 [.022, .054] |  | M3a-M2 | 19.133 (8)^*^ | -.008 | .004 |
| PC in school |  |  |  |  |  |  |  |  |  |  |
| M1. Configural model | 80.996 | 72 | .995 | .051 | .023 [.000, .045] |  |  |  |  |  |
| M2. Metric model | 89.143 | 80 | .995 | .068 | .022 [.000, .044] |  | M2-M1 | 7.850 (8) | .000 | -.001 |
| M3. Scalar model | 97.257 | 90 | .996 | .072 | .018 [.000, .040] |  | M3-M2 | 7.129 (10) | .001 | -.004 |
| PC out-of-school |  |  |  |  |  |  |  |  |  |  |
| M1. Configural model | 86.950 | 72 | .991 | .046 | .029 [.000, .050] |  |  |  |  |  |
| M2. Metric model | 96.405 | 80 | .990 | .065 | .029 [.000, .048] |  | M2-M1 | 9.421 (8) | -.001 | .000 |
| M3. Scalar model | 107.564 | 90 | .990 | .068 | .028 [.000, .047] |  | M3-M2 | 10.898 (10) | .000 | -.001 |
| NC in school |  |  |  |  |  |  |  |  |  |  |
| M1. Configural model | 126.747 | 72 | .958 | .043 | .056 [.040, .072] |  |  |  |  |  |
| M2. Metric model | 132.315 | 80 | .960 | .048 | .052 [.036, .068] |  | M2-M1 | 6.072 (8) | .002 | -.004 |
| M3. Scalar model | 152.525 | 90 | .952 | .053 | .054 [.039, .068] |  | M3-M2 | 20.669 (10)^*^ | -.008 | .002 |
| NC out-of-school |  |  |  |  |  |  |  |  |  |  |
| M1. Configural model | 87.335 | 72 | .988 | .040 | .030 [.000, .050] |  |  |  |  |  |
| M2. Metric model | 92.340 | 80 | .991 | .045 | .025 [.000, .046] |  | M2-M1 | 5.410 (8) | .003 | -.005 |
| M3. Scalar model | 123.673 | 90 | .974 | .063 | .039 [.020, .056] |  | M3-M2 | 36.176 (10)^***^ | -.017 | .014 |
| M3a. Partial scalar model^2^ | 105.831 | 86 | .985 | .051 | .031 [.000, .049] |  | M3a-M2 | 12.129 (6) | -.006 | .006 |

*Note.* M = Model; PC = Positive contact; NC = Negative contact; χ_SB_^2^ = Satorra-Bentler scaled chi-square; *df* = Degrees of Freedom; CFI = Comparative Fit Index; SRMR = Standardized Root Mean Square Residual; RMSEA = Root Mean Square Error of Approximation; CI = Confidence Interval; Δ = Change in the parameter.

^1^In this model, the intercepts of item 1 and item 2 were allowed to vary across time points. ^2^In this model, the intercepts of items 1 and 2 were allowed to vary across times.

^*^ *p* < .05; ^**^ *p* < .01, ^***^ *p* < .001

# Random Intercept Cross-Lagged Panel Models: Full Standardized Results

## Table S7

*Standardized Results of RI-CLPMs with Acculturation Preferences and Contact in School*

|  | Model A  Majority group | | | |  | Model B  Ethnic minority group | | | |
| --- | --- | --- | --- | --- | --- | --- | --- | --- | --- |
| WITHIN-PERSON Stability paths | T1→T2 | | T2→T3 | |  | T1→T2 | | T2→T3 | |
| Preference for maintenance (cen.) | .083 | | .078 | |  | .274 | | .282 | |
| Preference for maintenance (per.) | .030 | | .031 | |  | .057 | | .063 | |
| Preference for adoption (cen.) | .061 | | .058 | |  | .068 | | .076 | |
| Preference for adoption (per.) | .150 | | .146 | |  | -.018 | | -.019 | |
| Contact quantity | .160^*^ | | .191^*^ | |  | -.145 | | -.085 | |
| Positive contact | .187^*^ | | .211^*^ | |  | -.104 | | -.072 | |
| Negative contact | .217^*^ | | .220^*^ | |  | .028 | | .034 | |
| WITHIN-PERSON Cross-lagged paths | T1→T2 | | T2→T3 | |  | T1→T2 | | T2→T3 | |
| Preference for maintenance (cen.) → Preference for maintenance (per.) | .058 | | .060 | |  | .228 | | .254 | |
| Preference for maintenance (cen.) → Preference for adoption (cen.) | -.134^*^ | | -.128^*^ | |  | .109 | | .116 | |
| Preference for maintenance (cen.) → Preference for adoption (per.) | -.021 | | -.020 | |  | .026 | | .025 | |
| Preference for maintenance (cen.) → Contact quantity | -.029 | | -.028 | |  | -.094 | | -.063 | |
| Preference for maintenance (cen.) → Positive contact | .042 | | .039 | |  | -.012 | | -.009 | |
| Preference for maintenance (cen.) → Negative contact | -.049 | | -.042 | |  | -.226 | | -.224 | |
|  |  | |  | |  |  | |  | |
| Preference for maintenance (per.) → Preference for maintenance (cen.) | .001 | | .001 | |  | .143 | | .147 | |
| Preference for maintenance (per.) → Preference for adoption (cen.) | .021 | | .019 | |  | -.080 | | -.085 | |
| Preference for maintenance (per.) → Preference for adoption (per.) | -.010 | | -.010 | |  | -.170 | | -.161 | |
| Preference for maintenance (per.) → Contact quantity | .021 | | .020 | |  | .118 | | .079 | |
| Preference for maintenance (per.) → Positive contact | .058 | | .053 | |  | -.035 | | -.025 | |
| Preference for maintenance (per.) → Negative contact | -.017 | | -.014 | |  | .087 | | .086 | |
|  |  | |  | |  |  | |  | |
| Preference for adoption (cen.) → Preference for maintenance (cen.) | -.111 | | -.104 | |  | -.031 | | -.033 | |
| Preference for adoption (cen.) → Preference for maintenance (per.) | .018 | | .019 | |  | .021 | | .025 | |
| Preference for adoption (cen.) → Preference for adoption (per.) | .033 | | .032 | |  | -.048 | | -.048 | |
| Preference for adoption (cen.) → Contact quantity | .030 | | .029 | |  | -.208 | | -.145 | |
| Preference for adoption (cen.) → Positive contact | -.062 | | -.057 | |  | -.285 | | -.219 | |
| Preference for adoption (cen.) → Negative contact | -.088 | | -.076 | |  | -.092 | | -.095 | |
|  |  | |  | |  |  | |  | |
| Preference for adoption (per.) → Preference for maintenance (cen.) | -.045 | | -.043 | |  | -.144 | | -.164 | |
| Preference for adoption (per.) → Preference for maintenance (per.) | -.035 | | -.035 | |  | -.251^*^ | | -.309^*^ | |
| Preference for adoption (per.) → Preference for adoption (cen.) | .062 | | .060 | |  | -.188 | | -.222 | |
| Preference for adoption (per.) → Contact quantity | -.062 | | -.060 | |  | -.017 | | -.012 | |
| Preference for adoption (per.) → Positive contact | -.001 | | -.001 | |  | .160 | | .130 | |
| Preference for adoption (per.) → Negative contact | .055 | | .048 | |  | .224 | | .246 | |
|  |  | |  | |  |  | |  | |
| Contact quantity → Preference for maintenance (cen.) | .004 | | .005 | |  | -.047 | | -.043 | |
| Contact quantity → Preference for maintenance (per.) | -.029 | | -.038 | |  | .014 | | .014 | |
| Contact quantity → Preference for adoption (cen.) | .075 | | .090 | |  | -.017 | | -.016 | |
| Contact quantity → Preference for adoption (per.) | .004 | | .005 | |  | -.045 | | -.037 | |
| Contact quantity → Positive contact | .066 | | .077 | |  | -.276 | | -.180 | |
| Contact quantity → Negative contact | .017 | | .019 | |  | .048 | | .042 | |
|  |  | |  | |  |  | |  | |
| Positive contact → Preference for maintenance (cen.) | .055 | | .063 | |  | -.241^*^ | | -.233^*^ | |
| Positive contact → Preference for maintenance (per.) | .030 | | .038 | |  | -.199 | | -.208 | |
| Positive contact → Preference for adoption (cen.) | -.112^*^ | | -.129^*^ | |  | .004 | | .004 | |
| Positive contact → Preference for adoption (per.) | -.022 | | -.026 | |  | -.065 | | -.058 | |
| Positive contact → Contact quantity | .063 | | .072 | |  | -.235 | | -.147 | |
| Positive contact → Negative contact | -.051 | | -.054 | |  | .054 | | .050 | |
|  |  | |  | |  |  | |  | |
| Negative contact → Preference for maintenance (cen.) | -.032 | | -.036 | |  | -.084 | | -.107 | |
| Negative contact → Preference for maintenance (per.) | -.034 | | -.041 | |  | -.063 | | -.086 | |
| Negative contact → Preference for adoption (cen.) | .010 | | .011 | |  | -.002 | | -.003 | |
| Negative contact → Preference for adoption (per.) | .011 | | .012 | |  | .030 | | .035 | |
| Negative contact → Contact quantity | .025 | | .028 | |  | -.004 | | -.003 | |
| Negative contact → Positive contact | .009 | | .009 | |  | -.056 | | -.051 | |
| WITHIN-PERSON Correlations and Residual Covariances | T1 | T2 | | T3 |  | T1 | T2 | | T3 |
| Preference for maintenance (cen.) ↔ Preference for maintenance (per.) | .369^***^ | .389^***^ | | .455^***^ |  | .771^***^ | .724^***^ | | .823^***^ |
| Preference for maintenance (cen.) ↔ Preference for adoption (cen.) | -.187^*^ | -.147^*^ | | -.156^*^ |  | -.084 | -.075 | | .057 |
| Preference for maintenance (cen.) ↔ Preference for adoption (per.) | -.059 | -.001 | | -.001 |  | -.193 | -.035 | | -.033 |
| Preference for maintenance (cen.) ↔ Contact quantity | -.046 | .046 | | .049 |  | .068 | -.190 | | -.122 |
| Preference for maintenance (cen.) ↔ Positive contact | .115 | .121^*^ | | .126^*^ |  | -.251 | -.185 | | -.133 |
| Preference for maintenance (cen.) ↔ Negative contact | -.079 | -.066 | | -.064 |  | -.269 | .003 | | .180^*^ |
| Preference for maintenance (per.) ↔ Preference for adoption (cen.) | .022 | .087 | | .103 |  | -.106 | -.020 | | -.025 |
| Preference for maintenance (per.) ↔ Preference for adoption (per.) | .041 | .018 | | .021 |  | -.142 | -.125 | | -.133 |
| Preference for maintenance (per.) ↔ Contact quantity | .014 | .060 | | .072 |  | .120 | -.046 | | -.033 |
| Preference for maintenance (per.) ↔ Positive contact | -.001 | .047 | | .055 |  | -.175 | -.153 | | -.123 |
| Preference for maintenance (per.) ↔ Negative contact | -.012 | -.060 | | -.065 |  | -.109 | .143 | | .157 |
| Preference for adoption (cen.) ↔ Preference for adoption (per.) | .567^***^ | .449^***^ | | .488^***^ |  | .455^**^ | .557^**^ | | .556^**^ |
| Preference for adoption (cen.) ↔ Contact quantity | .127 | .023 | | .025 |  | -.110 | -.081 | | -.055 |
| Preference for adoption (cen.) ↔ Positive contact | -.031 | -.036 | | -.038 |  | -.109 | .011 | | .009 |
| Preference for adoption (cen.) ↔ Negative contact | .121 | .075 | | .073 |  | -.051 | .074 | | .012 |
| Preference for adoption (per.) ↔ Contact quantity | .076 | .039 | | .042 |  | .274^*^ | -.015 | | -.009 |
| Preference for adoption (per.) ↔ Positive contact | .107 | -.001 | | -.002 |  | .117 | .142 | | .095 |
| Preference for adoption (per.) ↔ Negative contact | -.006 | .024 | | .023 |  | .102 | .013 | | .012 |
| Contact quantity ↔ Positive contact | .338^***^ | .404^***^ | | .431^***^ |  | .246 | .177 | | .579^***^ |
| Contact quantity ↔ Negative contact | -.076 | -.013 | | -.013 |  | .164 | .040 | | -.306^*^ |
| Positive contact ↔ Negative contact | -.399^**^ | -.313^***^ | | -.303^***^ |  | -.251 | -.174 | | -.570^***^ |
| BETWEEN-PERSON Correlations |  | | | |  |  | | | |
| Preference for maintenance (cen.) ↔ Preference for maintenance (per.) | .629^**^ | | | |  | .155 | | | |
| Preference for maintenance (cen.) ↔ Preference for adoption (cen.) | -.433^*^ | | | |  | .275 | | | |
| Preference for maintenance (cen.) ↔ Preference for adoption (per.) | -.184 | | | |  | .428 | | | |
| Preference for maintenance (cen.) ↔ Contact quantity | .167 | | | |  | -.215 | | | |
| Preference for maintenance (cen.) ↔ Positive contact | .356^**^ | | | |  | .101 | | | |
| Preference for maintenance (cen.) ↔ Negative contact | -.388^**^ | | | |  | .942 | | | |
| Preference for maintenance (per.) ↔ Preference for adoption (cen.) | -.013 | | | |  | .363 | | | |
| Preference for maintenance (per.) ↔ Preference for adoption (per.) | -.159 | | | |  | .511 | | | |
| Preference for maintenance (per.) ↔ Contact quantity | .101 | | | |  | -.424 | | | |
| Preference for maintenance (per.) ↔ Positive contact | .301^**^ | | | |  | .183 | | | |
| Preference for maintenance (per.) ↔ Negative contact | -.182 | | | |  | .550 | | | |
| Preference for adoption (cen.) ↔ Preference for adoption (per.) | .821^***^ | | | |  | .793^***^ | | | |
| Preference for adoption (cen.) ↔ Contact quantity | -.117 | | | |  | .389 | | | |
| Preference for adoption (cen.) ↔ Positive contact | -.359^**^ | | | |  | .496^**^ | | | |
| Preference for adoption (cen.) ↔ Negative contact | .532^***^ | | | |  | -.199 | | | |
| Preference for adoption (per.) ↔ Contact quantity | -.023 | | | |  | .675^*^ | | | |
| Preference for adoption (per.) ↔ Positive contact | -.370^**^ | | | |  | .612^**^ | | | |
| Preference for adoption (per.) ↔ Negative contact | .513^**^ | | | |  | -.610^*^ | | | |
| Contact quantity ↔ Positive contact | .490^***^ | | | |  | .693^***^ | | | |
| Contact quantity ↔ Negative contact | -.182 | | | |  | -.562 | | | |
| Positive contact ↔ Negative contact | -.800^***^ | | | |  | -.955^***^ | | | |

*Note:* cen. = Central domains; per. = Peripheral domains; *T* = time;

^*^ *p* < .05; ^**^ *p* < .01, ^***^ *p* < .001

## Table S8

*Standardized Results of RI-CLPMs with Acculturation Preferences and Contact Out-of-School*

|  | Model C  Majority group | | | |  | Model D  Ethnic minority group | | | |
| --- | --- | --- | --- | --- | --- | --- | --- | --- | --- |
| WITHIN-PERSON Stability paths | T1→T2 | | T2→T3 | |  | T1→T2 | | T2→T3 | |
| Preference for maintenance (cen.) | .102 | | .097 | |  | .403 | | .417 | |
| Preference for maintenance (per.) | .028 | | .030 | |  | .000 | | .000 | |
| Preference for adoption (cen.) | .136 | | .002 | |  | .167 | | .187 | |
| Preference for adoption (per.) | .157 | | .151^*^ | |  | -.018 | | -.019 | |
| Contact quantity | -.092 | | .054 | |  | .104 | | .107 | |
| Positive contact | .161 | | .133 | |  | -.121 | | -.070 | |
| Negative contact | .130 | | .126 | |  | -.069 | | -.101 | |
| WITHIN-PERSON Cross-lagged paths | T1→T2 | | T2→T3 | |  | T1→T2 | | T2→T3 | |
| Preference for maintenance (cen.) → Preference for maintenance (per.) | .066 | | .070 | |  | .268 | | .290 | |
| Preference for maintenance (cen.) → Preference for adoption (cen.) | -.160^*^ | | -.156^*^ | |  | .083 | | .089 | |
| Preference for maintenance (cen.) → Preference for adoption (per.) | -.043 | | -.042 | |  | .053 | | .050 | |
| Preference for maintenance (cen.) → Contact quantity | .094 | | .088 | |  | .005 | | .004 | |
| Preference for maintenance (cen.) → Positive contact | .005 | | .004 | |  | -.179 | | -.120 | |
| Preference for maintenance (cen.) → Negative contact | -.026 | | -.023 | |  | -.309 | | -.321 | |
|  |  | |  | |  |  | |  | |
| Preference for maintenance (per.) → Preference for maintenance (cen.) | .000 | | .000 | |  | .049 | | .050 | |
| Preference for maintenance (per.) → Preference for adoption (cen.) | .031 | | .030 | |  | -.062 | | -.065 | |
| Preference for maintenance (per.) → Preference for adoption (per.) | -.009 | | -.008 | |  | -.157 | | -.147 | |
| Preference for maintenance (per.) → Contact quantity | .011 | | .011 | |  | .124 | | .110 | |
| Preference for maintenance (per.) → Positive contact | .092 | | .080 | |  | .162 | | .106 | |
| Preference for maintenance (per.) → Negative contact | -.055 | | -.048 | |  | .149 | | .151 | |
|  |  | |  | |  |  | |  | |
| Preference for adoption (cen.) → Preference for maintenance (cen.) | -.119 | | -.110 | |  | -.048 | | -.052 | |
| Preference for adoption (cen.) → Preference for maintenance (per.) | .012 | | .012 | |  | -.025 | | -.028 | |
| Preference for adoption (cen.) → Preference for adoption (per.) | .024 | | .023 | |  | -.032 | | -.032 | |
| Preference for adoption (cen.) → Contact quantity | -.070 | | -.063 | |  | -.092 | | -.087 | |
| Preference for adoption (cen.) → Positive contact | -.107 | | -.091 | |  | -.161 | | -.111 | |
| Preference for adoption (cen.) → Negative contact | -.118 | | -.100 | |  | -.030 | | -.032 | |
|  |  | |  | |  |  | |  | |
| Preference for adoption (per.) → Preference for maintenance (cen.) | -.050 | | -.047 | |  | -.141 | | -.160 | |
| Preference for adoption (per.) → Preference for maintenance (per.) | -.029 | | -.031 | |  | -.253^*^ | | -.300^*^ | |
| Preference for adoption (per.) → Preference for adoption (cen.) | .046 | | .045 | |  | -.245^*^ | | -.290^*^ | |
| Preference for adoption (per.) → Contact quantity | .032 | | .029 | |  | -.116 | | -.115 | |
| Preference for adoption (per.) → Positive contact | .005 | | .004 | |  | -.062 | | -.046 | |
| Preference for adoption (per.) → Negative contact | .036 | | .031 | |  | -.105 | | -.120 | |
|  |  | |  | |  |  | |  | |
| Contact quantity → Preference for maintenance (cen.) | .013 | | .012 | |  | .082 | | .096 | |
| Contact quantity → Preference for maintenance (per.) | .009 | | .010 | |  | .105 | | .128 | |
| Contact quantity → Preference for adoption (cen.) | -.083 | | -.083 | |  | -.077 | | -.093 | |
| Contact quantity → Preference for adoption (per.) | -.051 | | -.050 | |  | -.031 | | -.033 | |
| Contact quantity → Positive contact | .011 | | .010 | |  | -.165 | | -.125 | |
| Contact quantity → Negative contact | -.036 | | -.033 | |  | -.063 | | -.074 | |
|  |  | |  | |  |  | |  | |
| Positive contact → Preference for maintenance (cen.) | .048 | | .043 | |  | -.133 | | -.118 | |
| Positive contact → Preference for maintenance (per.) | .087 | | .087 | |  | -.013 | | -.012 | |
| Positive contact → Preference for adoption (cen.) | .057 | | .053 | |  | -.141 | | -.131 | |
| Positive contact → Preference for adoption (per.) | .065 | | .060 | |  | -.230 | | -.189 | |
| Positive contact → Contact quantity | .249^**^ | | -.061 | |  | -.198 | | -.154 | |
| Positive contact → Negative contact | -.003 | | -.002 | |  | .082 | | .073 | |
|  |  | |  | |  |  | |  | |
| Negative contact → Preference for maintenance (cen.) | -.014 | | -.015 | |  | -.107 | | -.157 | |
| Negative contact → Preference for maintenance (per.) | -.064 | | -.076 | |  | -.036 | | -.055 | |
| Negative contact → Preference for adoption (cen.) | -.048 | | -.052 | |  | -.159 | | -.244 | |
| Negative contact → Preference for adoption (per.) | .021 | | .023 | |  | -.231^*^ | | -.315^*^ | |
| Negative contact → Contact quantity | -.099 | | -.102 | |  | -.117 | | -.151 | |
| Negative contact → Positive contact | -.036 | | -.035 | |  | -.198 | | -.188 | |
| WITHIN-PERSON Correlations and Residual Covariances | T1 | T2 | | T3 |  | T1 | T2 | | T3 |
| Preference for maintenance (cen.) ↔ Preference for maintenance (per.) | .379^***^ | .388^***^ | | .459^***^ |  | .747^***^ | .722^***^ | | .820^***^ |
| Preference for maintenance (cen.) ↔ Preference for adoption (cen.) | -.203^*^ | -.154^*^ | | -.164^*^ |  | -.078 | -.148 | | -.002 |
| Preference for maintenance (cen.) ↔ Preference for adoption (per.) | -.083 | -.010 | | -.011 |  | -.095 | -.029 | | -.029 |
| Preference for maintenance (cen.) ↔ Contact quantity | .173^*^ | .078 | | .078 |  | .313 | -.015 | | -.014 |
| Preference for maintenance (cen.) ↔ Positive contact | .169^*^ | .117^*^ | | .113^*^ |  | -.103 | -.129 | | -.086 |
| Preference for maintenance (cen.) ↔ Negative contact | -.019 | .009 | | .008 |  | -.228 | -.022 | | -.023 |
| Preference for maintenance (per.) ↔ Preference for adoption (cen.) | .018 | .076 | | .092 |  | -.133 | -.054 | | -.064 |
| Preference for maintenance (per.) ↔ Preference for adoption (per.) | .028 | .012 | | .014 |  | -.094 | -.111 | | -.115 |
| Preference for maintenance (per.) ↔ Contact quantity | .164^*^ | .098^*^ | | .111^*^ |  | .317^*^ | .008 | | .008 |
| Preference for maintenance (per.) ↔ Positive contact | .152^*^ | .134^**^ | | .145^**^ |  | .006 | -.143 | | -.101 |
| Preference for maintenance (per.) ↔ Negative contact | -.069 | -.027 | | -.030 |  | -.027 | .018 | | .021 |
| Preference for adoption (cen.) ↔ Preference for adoption (per.) | .573^***^ | .434^***^ | | .474^***^ |  | .459^**^ | .492^**^ | | .496^**^ |
| Preference for adoption (cen.) ↔ Contact quantity | -.025 | -.011 | | -.011 |  | -.103 | -.194 | | -.189^*^ |
| Preference for adoption (cen.) ↔ Positive contact | -.061 | .010 | | .010 |  | -.152 | .149 | | .066 |
| Preference for adoption (cen.) ↔ Negative contact | .079 | -.011 | | -.011 |  | .016 | -.029 | | -.115 |
| Preference for adoption (per.) ↔ Contact quantity | -.038 | .020 | | .021 |  | -.010 | -.083 | | -.071 |
| Preference for adoption (per.) ↔ Positive contact | -.118 | -.024 | | -.023 |  | -.049 | -.149 | | -.090 |
| Preference for adoption (per.) ↔ Negative contact | .084 | .025 | | .024 |  | -.050 | -.117 | | -.115 |
| Contact quantity ↔ Positive contact | .561^***^ | .504^***^ | | .464^***^ |  | .385^**^ | .074 | | .388^**^ |
| Contact quantity ↔ Negative contact | -.078 | -.109 | | -.101 |  | -.111 | -.404^***^ | | -.380^***^ |
| Positive contact ↔ Negative contact | -.247^***^ | -.328^**^ | | -.293^**^ |  | -.056 | -.321^**^ | | -.492^***^ |
| BETWEEN-PERSON Correlations |  | | | |  |  | | | |
| Preference for maintenance (cen.) ↔ Preference for maintenance (per.) | .624^***^ | | | |  | .368 | | | |
| Preference for maintenance (cen.) ↔ Preference for adoption (cen.) | -.425^***^ | | | |  | .445 | | | |
| Preference for maintenance (cen.) ↔ Preference for adoption (per.) | -.153 | | | |  | .203 | | | |
| Preference for maintenance (cen.) ↔ Contact quantity | .019 | | | |  | -1.039 | | | |
| Preference for maintenance (cen.) ↔ Positive contact | .303^*^ | | | |  | .029 | | | |
| Preference for maintenance (cen.) ↔ Negative contact | -.352^**^ | | | |  | 1.189 | | | |
| Preference for maintenance (per.) ↔ Preference for adoption (cen.) | -.011 | | | |  | .404 | | | |
| Preference for maintenance (per.) ↔ Preference for adoption (per.) | -.139 | | | |  | .316 | | | |
| Preference for maintenance (per.) ↔ Contact quantity | -.010 | | | |  | -.628 | | | |
| Preference for maintenance (per.) ↔ Positive contact | .069 | | | |  | -.011 | | | |
| Preference for maintenance (per.) ↔ Negative contact | -.133 | | | |  | .399 | | | |
| Preference for adoption (cen.) ↔ Preference for adoption (per.) | .832^***^ | | | |  | .864^***^ | | | |
| Preference for adoption (cen.) ↔ Contact quantity | -.072 | | | |  | .670^**^ | | | |
| Preference for adoption (cen.) ↔ Positive contact | -.289^*^ | | | |  | .598^**^ | | | |
| Preference for adoption (cen.) ↔ Negative contact | .493^***^ | | | |  | -.083 | | | |
| Preference for adoption (per.) ↔ Contact quantity | .085 | | | |  | .949^**^ | | | |
| Preference for adoption (per.) ↔ Positive contact | -.178 | | | |  | .735^***^ | | | |
| Preference for adoption (per.) ↔ Negative contact | .379^*^ | | | |  | -.200 | | | |
| Contact quantity ↔ Positive contact | .579^***^ | | | |  | .906^***^ | | | |
| Contact quantity ↔ Negative contact | .124 | | | |  | -.078 | | | |
| Positive contact ↔ Negative contact | -.576^***^ | | | |  | -.804^***^ | | | |

*Note:* cen. = Central domains; per. = Peripheral domains; *T* = time;

^*^ *p* < .05; ^**^ *p* < .01, ^***^ *p* < .001

# Sensitivity Analyses: Random Intercept Cross-Lagged Panel Models with *Type=Complex*

As additional sensitivity analyses, the four Random-Intercept Cross-Lagged Panel Models were tested again using the *Type=Complex* function available in M*plus*. This function provides robust standard errors that account for the nested structure of the data. Specifically, we accounted for the fact that participating adolescents were nested in 48 classrooms^[1]^. The procedure followed is identical to the one reported in the main text. Of note, all models with *Type=Complex* resulted in warnings due to the fact that the number of parameters was higher the than the number of clusters (i.e., classrooms). Thus, results should be interpreted with caution.

Model fit and model comparisons of these sensitivity RI-CLPM are reported in Table S9. Full standardized results are presented in Table S10 (acculturation and contact in school for majority and ethnic minority adolescents, separately) and in Table S11 (acculturation and contact out-of-school for majority and ethnic minority adolescents, separately). Findings from all these models largely replicated those obtained without *Type=Complex.*

For what concerns models on acculturation preferences and contact in school, results were fully replicated for the ethnic minority and majority groups, albeit for the latter the within-person autoregression of positive contact lost significance (*p* =.066). Models on acculturation preferences and contact out-of-school showed a few differences compared to main findings. For the majority group, the within-person association from preference for maintenance and preference for adoption in central domains became not significant as well as their concurrent association at T2. Additionally, within-person increases in preference for destination culture adoption in central domain contributed to within-person decreases in negative contact experiences at the following time. For the ethnic minority group, accounting for the nested structure of the data allowed to reach only partial invariance of cross-lagged paths (contrary to the main analyses, where full invariance was reached). As a consequence, in this model the within-person cross-lagged path from preference for adoption in peripheral domains and preference for maintenance in peripheral domains lost significance between T1 and T2 (but not at the following wave). Moreover, adolescents who showed increases in positive out-of-school contact at one time point reported decreases in the frequency of contact later on, an association that was not significant in the main model. Overall, limited differences emerged between the main and sensitivity models and they mostly did not involve concurrent and longitudinal associations between acculturation preferences and contact.

## Table S9

*Random Intercept Cross-Lagged Panel Models with* Type=Complex*: Model fit and comparisons*

|  | Model fit | | | | | Model comparisons | | | |
| --- | --- | --- | --- | --- | --- | --- | --- | --- | --- |
| Models | χ_SB_^2^ | df | CFI | SRMR | RMSEA [90% CI] | Models | Δχ_SB_^2^ | ΔCFI | ΔRMSEA |
| **Majority’s acculturation preferences and contact in school** | | | | | | | | | |
| Unconstrained (M1) | 30.569 | 21 | .997 | .016 | .025 [.000, .043] |  |  |  |  |
| Autoregressions fixed (M2) | 51.977 | 28 | .994 | .023 | .034 [.019, .048] | M2-M1 | 20.541 (7)^**^ | -.003 | .009 |
| Autoregressions and cross-lagged paths fixed (M3) | 107.800 | 70 | .990 | .036 | .027 [.016, .037] | M3-M2 | 56.944 (42) | -.004 | -.007 |
| Autoregressions, cross-lagged paths, and residual covariances fixed (M4) | 143.911 | 91 | .986 | .042 | .028 [.019, .036] | M4-M3 | 35.633 (21)^*^ | -.004 | .001 |
| **Ethnic minority’s acculturation preferences and contact in school** | | | | | | | | | |
| Unconstrained (M1) | 29.650 | 21 | .994 | .021 | .041 [.000, .073] |  |  |  |  |
| Autoregressions fixed (M2) | 45.686 | 28 | .987 | .026 | .051 [.021, .077] | M2-M1 | 15.635 (7)^*^ | -.007 | .010 |
| Autoregressions and cross-lagged paths fixed (M3) | 86.129 | 70 | .988 | .048 | .031 [.000, .051] | M3-M2 | 42.634 (42) | -.001 | -.020 |
| Autoregressions, cross-lagged paths, and residual covariances fixed (M4) | 137.005 | 91 | .967 | .083 | .046 [.029, .061] | M4-M3 | 45.308 (21)^**^ | -.021 | .015 |
| Autoregressions, cross-lagged paths, and residual covariances partially fixed (M4a)^1^ | 114.299 | 86 | .980 | .061 | .037 [.015, .054] | M4a-M3 | 26.665 (16) ^*^ | -.008 | .006 |
| **Majority’s acculturation preferences and contact out-of-school** | | | | | | | | | |
| Unconstrained (M1) | 10.293 | 21 | 1.000 | .009 | .000 [.000, .000] |  |  |  |  |
| Autoregressions fixed (M2) | 36.646 | 28 | .998 | .018 | .020 [.000, .037] | M2-M1 | 29.555 (7)^***^ | -.002 | .020 |
| Autoregressions partially fixed (M2a)^2^ | 20.910 | 26 | 1.000 | .013 | .000 [.000, .021] | M2a-M1 | 10.591 (5) | .000 | .000 |
| Autoregressions and cross-lagged paths fixed (M3) | 84.780 | 68 | .996 | .031 | .018 [.000, .030] | M3-M2a | 63.116 (42)^*^ | -.004 | .018 |
| Autoregressions and cross-lagged paths partially fixed (M3a)^3^ | 71.740 | 67 | .999 | .029 | .010 [.000, .024] | M3a-M2a | 50.183 (41) | -.001 | .010 |
| Autoregressions, cross-lagged paths and residual covariances fixed (M4) | 95.172 | 88 | .998 | .035 | .011 [.000, .023] | M4-M3a | 23.360 (21) | -.001 | .001 |
| **Ethnic minority’s acculturation preferences and contact out-of-school** | | | | | | | | | |
| Unconstrained (M1) | 48.370 | 21 | .978 | .037 | .074 [.046, .101] |  |  |  |  |
| Autoregressions fixed (M2) | 51.622 | 28 | .981 | .043 | .059 [.033, .084] | M2-M1 | 7.737 (7) | .003 | -.015 |
| Autoregressions and cross-lagged paths fixed (M3) | 79.017 | 70 | .993 | .072 | .023 [.000, .046] | M3-M2 | 34.751 (42) | .012 | -.036 |
| Autoregressions and cross-lagged paths partially fixed (M3a)^4^ | 79.343 | 67 | .990 | .072 | .028 [.000, .049] | M3a-M2 | 34.267 (39) | .009 | -.031 |
| Autoregressions, cross-lagged paths, and residual covariances fixed (M4) | 120.110 | 88 | .974 | .078 | .039 [.019, .055] | M4-M3 | 39.820 (21)^**^ | -.016 | .011 |
| Autoregressions, cross-lagged paths, and residual covariances partially fixed (M4a)^5^ | 102.525 | 85 | .986 | .075 | .029 [.000, .048] | M4a-M3 | 23.106 (18) | -.004 | .001 |

*Note*. M = Model; χ2 = Chi-square; df = Degree of Freedom; CFI = Comparative Fit Index; SRMR = Standardized Root Mean Square Residual; RMSEA = Root Mean Square Error of Approximation; CI = Confidence Interval; Δ = Change in the parameter. ^1^In this model, the following residual covariances were freed: between maintenance and adoption in central domains, between maintenance in central domains and negative contact, between quantity, positive, and negative contact. ^2^In this model, the autoregression paths of adoption in central domains and contact quantity were freed. ^3^In this model, the cross-lagged path from positive contact to contact quantity was freed. ^4^In this model, the following cross-lagged associations were freed: from preference for adoption to maintenance in peripheral domains, from preference for maintenance in central domains to adoption in peripheral ones, and from adoption in central domains to quantity of contact. ^5^In this model, the following residual covariances were freed: between maintenance and adoption in central domains, between quantity and positive contact, and between positive and negative contact.

^*^ *p* < .05; ^**^ *p* < .01, ^***^ *p* < .001

## Table S10

*Standardized Results of RI-CLPMs with* Type=Complex*: Acculturation Preferences and Contact in School*

|  | Majority group | | | |  | Ethnic minority group | | | |
| --- | --- | --- | --- | --- | --- | --- | --- | --- | --- |
| WITHIN-PERSON Stability paths | T1→T2 | | T2→T3 | |  | T1→T2 | | T2→T3 | |
| Preference for maintenance (cen.) | .083 | | .078 | |  | .274 | | .282 | |
| Preference for maintenance (per.) | .030 | | .031 | |  | .057 | | .063 | |
| Preference for adoption (cen.) | .061 | | .058 | |  | .068 | | .076 | |
| Preference for adoption (per.) | .150 | | .146 | |  | -.018 | | -.019 | |
| Contact quantity | .160^*^ | | .191^*^ | |  | -.145 | | -.085 | |
| Positive contact | .187 | | .211 | |  | -.104 | | -.072 | |
| Negative contact | .217^*^ | | .220^*^ | |  | .028 | | .034 | |
| WITHIN-PERSON Cross-lagged paths | T1→T2 | | T2→T3 | |  | T1→T2 | | T2→T3 | |
| Preference for maintenance (cen.) → Preference for maintenance (per.) | .058 | | .060 | |  | .228 | | .254 | |
| Preference for maintenance (cen.) → Preference for adoption (cen.) | -.134^*^ | | -.128^*^ | |  | .109 | | .116 | |
| Preference for maintenance (cen.) → Preference for adoption (per.) | -.021 | | -.020 | |  | .026 | | .025 | |
| Preference for maintenance (cen.) → Contact quantity | -.029 | | -.028 | |  | -.094 | | -.063 | |
| Preference for maintenance (cen.) → Positive contact | .042 | | .039 | |  | -.012 | | -.009 | |
| Preference for maintenance (cen.) → Negative contact | -.049 | | -.042 | |  | -.226 | | -.224 | |
|  |  | |  | |  |  | |  | |
| Preference for maintenance (per.) → Preference for maintenance (cen.) | .001 | | .001 | |  | .143 | | .147 | |
| Preference for maintenance (per.) → Preference for adoption (cen.) | .021 | | .019 | |  | -.080 | | -.085 | |
| Preference for maintenance (per.) → Preference for adoption (per.) | -.010 | | -.010 | |  | -.170 | | -.161 | |
| Preference for maintenance (per.) → Contact quantity | .021 | | .020 | |  | .118 | | .079 | |
| Preference for maintenance (per.) → Positive contact | .058 | | .053 | |  | -.035 | | -.025 | |
| Preference for maintenance (per.) → Negative contact | -.017 | | -.014 | |  | .087 | | .086 | |
|  |  | |  | |  |  | |  | |
| Preference for adoption (cen.) → Preference for maintenance (cen.) | -.111 | | -.104 | |  | -.031 | | -.033 | |
| Preference for adoption (cen.) → Preference for maintenance (per.) | .018 | | .019 | |  | .021 | | .025 | |
| Preference for adoption (cen.) → Preference for adoption (per.) | .033 | | .032 | |  | -.048 | | -.048 | |
| Preference for adoption (cen.) → Contact quantity | .030 | | .029 | |  | -.208 | | -.145 | |
| Preference for adoption (cen.) → Positive contact | -.062 | | -.057 | |  | -.285 | | -.219 | |
| Preference for adoption (cen.) → Negative contact | -.088 | | -.076 | |  | -.092 | | -.095 | |
|  |  | |  | |  |  | |  | |
| Preference for adoption (per.) → Preference for maintenance (cen.) | -.045 | | -.043 | |  | -.144 | | -.164 | |
| Preference for adoption (per.) → Preference for maintenance (per.) | -.032 | | -.034 | |  | -.251^*^ | | -.309^*^ | |
| Preference for adoption (per.) → Preference for adoption (cen.) | .062 | | .060 | |  | -.188 | | -.222 | |
| Preference for adoption (per.) → Contact quantity | -.062 | | -.060 | |  | -.017 | | -.012 | |
| Preference for adoption (per.) → Positive contact | -.001 | | -.001 | |  | .160 | | .130 | |
| Preference for adoption (per.) → Negative contact | .055 | | .048 | |  | .224 | | .246 | |
|  |  | |  | |  |  | |  | |
| Contact quantity → Preference for maintenance (cen.) | .004 | | .005 | |  | -.047 | | -.043 | |
| Contact quantity → Preference for maintenance (per.) | -.029 | | -.038 | |  | .014 | | .014 | |
| Contact quantity → Preference for adoption (cen.) | .075 | | .090 | |  | -.017 | | -.016 | |
| Contact quantity → Preference for adoption (per.) | .004 | | .005 | |  | -.045 | | -.037 | |
| Contact quantity → Positive contact | .066 | | .077 | |  | -.276 | | -.180 | |
| Contact quantity → Negative contact | .017 | | .019 | |  | .048 | | .042 | |
|  |  | |  | |  |  | |  | |
| Positive contact → Preference for maintenance (cen.) | .055 | | .063 | |  | -.241^*^ | | -.233^*^ | |
| Positive contact → Preference for maintenance (per.) | .030 | | .038 | |  | -.199 | | -.208 | |
| Positive contact → Preference for adoption (cen.) | -.112^*^ | | -.129^*^ | |  | .004 | | .004 | |
| Positive contact → Preference for adoption (per.) | -.022 | | -.026 | |  | -.065 | | -.058 | |
| Positive contact → Contact quantity | .063 | | .072 | |  | -.235 | | -.147 | |
| Positive contact → Negative contact | -.051 | | -.054 | |  | .054 | | .050 | |
|  |  | |  | |  |  | |  | |
| Negative contact → Preference for maintenance (cen.) | -.032 | | -.036 | |  | -.084 | | -.107 | |
| Negative contact → Preference for maintenance (per.) | -.034 | | -.041 | |  | -.063 | | -.086 | |
| Negative contact → Preference for adoption (cen.) | .010 | | .011 | |  | -.002 | | -.003 | |
| Negative contact → Preference for adoption (per.) | .011 | | .012 | |  | .030 | | .035 | |
| Negative contact → Contact quantity | .025 | | .028 | |  | -.004 | | -.003 | |
| Negative contact → Positive contact | .009 | | .009 | |  | -.056 | | -.051 | |
| WITHIN-PERSON Correlations and Residual Covariances | T1 | T2 | | T3 |  | T1 | T2 | | T3 |
| Preference for maintenance (cen.) ↔ Preference for maintenance (per.) | .369^***^ | .389^***^ | | .455^***^ |  | .771^***^ | .724^***^ | | .823^***^ |
| Preference for maintenance (cen.) ↔ Preference for adoption (cen.) | -.187^*^ | -.147^*^ | | -.156^*^ |  | -.084 | -.075 | | .057 |
| Preference for maintenance (cen.) ↔ Preference for adoption (per.) | -.059 | -.001 | | -.001 |  | -.193 | -.035 | | -.033 |
| Preference for maintenance (cen.) ↔ Contact quantity | -.046 | .046 | | .049 |  | .068 | -.190 | | -.122 |
| Preference for maintenance (cen.) ↔ Positive contact | .115 | .121^*^ | | .126^*^ |  | -.251 | -.185 | | -.133 |
| Preference for maintenance (cen.) ↔ Negative contact | -.079 | -.066 | | -.064 |  | -.269 | .003 | | .180^*^ |
| Preference for maintenance (per.) ↔ Preference for adoption (cen.) | .022 | .087 | | .103 |  | -.106 | -.020 | | -.025 |
| Preference for maintenance (per.) ↔ Preference for adoption (per.) | .041 | .018 | | .021 |  | -.142 | -.125 | | -.133 |
| Preference for maintenance (per.) ↔ Contact quantity | .014 | .060 | | .072 |  | .120 | -.046 | | -.033 |
| Preference for maintenance (per.) ↔ Positive contact | -.001 | .047 | | .055 |  | -.175 | -.153 | | -.123 |
| Preference for maintenance (per.) ↔ Negative contact | -.012 | -.060 | | -.065 |  | -.109 | .143 | | .157 |
| Preference for adoption (cen.) ↔ Preference for adoption (per.) | .567^***^ | .449^***^ | | .488^***^ |  | .455^***^ | .557^**^ | | .556^**^ |
| Preference for adoption (cen.) ↔ Contact quantity | .127 | .023 | | .025 |  | -.110 | -.081 | | -.055 |
| Preference for adoption (cen.) ↔ Positive contact | -.031 | -.036 | | -.038 |  | -.109 | .011 | | .009 |
| Preference for adoption (cen.) ↔ Negative contact | .121 | .075 | | .073 |  | -.051 | .074 | | .076 |
| Preference for adoption (per.) ↔ Contact quantity | .076 | .039 | | .042 |  | .274^*^ | -.015 | | -.009 |
| Preference for adoption (per.) ↔ Positive contact | .107 | -.001 | | -.002 |  | .117 | .142 | | .095 |
| Preference for adoption (per.) ↔ Negative contact | -.006 | .024 | | .023 |  | .102 | .013 | | .012 |
| Contact quantity ↔ Positive contact | .338^***^ | .404^***^ | | .431^***^ |  | .246 | .177 | | .579^***^ |
| Contact quantity ↔ Negative contact | -.076 | -.013 | | -.013 |  | .164 | .040 | | -.306 |
| Positive contact ↔ Negative contact | -.399^***^ | -.313^***^ | | -.303^***^ |  | -.251 | -.174 | | -.570^***^ |
| BETWEEN-PERSON Correlations |  | | | |  |  | | | |
| Preference for maintenance (cen.) ↔ Preference for maintenance (per.) | .629^**^ | | | |  | .155 | | | |
| Preference for maintenance (cen.) ↔ Preference for adoption (cen.) | -.433^**^ | | | |  | .275 | | | |
| Preference for maintenance (cen.) ↔ Preference for adoption (per.) | -.184 | | | |  | .428 | | | |
| Preference for maintenance (cen.) ↔ Contact quantity | .167 | | | |  | -.215 | | | |
| Preference for maintenance (cen.) ↔ Positive contact | .356^**^ | | | |  | .101 | | | |
| Preference for maintenance (cen.) ↔ Negative contact | -.388^**^ | | | |  | .942 | | | |
| Preference for maintenance (per.) ↔ Preference for adoption (cen.) | -.013 | | | |  | .363 | | | |
| Preference for maintenance (per.) ↔ Preference for adoption (per.) | -.159 | | | |  | .511 | | | |
| Preference for maintenance (per.) ↔ Contact quantity | .101 | | | |  | -.424 | | | |
| Preference for maintenance (per.) ↔ Positive contact | .301^**^ | | | |  | .183 | | | |
| Preference for maintenance (per.) ↔ Negative contact | -.182 | | | |  | .550 | | | |
| Preference for adoption (cen.) ↔ Preference for adoption (per.) | .821^***^ | | | |  | .793^***^ | | | |
| Preference for adoption (cen.) ↔ Contact quantity | -.117 | | | |  | .389 | | | |
| Preference for adoption (cen.) ↔ Positive contact | -.359^**^ | | | |  | .496^**^ | | | |
| Preference for adoption (cen.) ↔ Negative contact | .532^***^ | | | |  | -.199 | | | |
| Preference for adoption (per.) ↔ Contact quantity | -.023 | | | |  | .675^*^ | | | |
| Preference for adoption (per.) ↔ Positive contact | -.370^**^ | | | |  | .612^***^ | | | |
| Preference for adoption (per.) ↔ Negative contact | .513^**^ | | | |  | -.610^*^ | | | |
| Contact quantity ↔ Positive contact | .490^***^ | | | |  | .693^***^ | | | |
| Contact quantity ↔ Negative contact | -.182 | | | |  | -.562 | | | |
| Positive contact ↔ Negative contact | -.800^***^ | | | |  | -.955^***^ | | | |

*Note:* cen. = Central domains; per. = Peripheral domains; *T* = time. Grey-shaded values lost significance when accounting for the nested structure of the data.

^*^ *p* < .05; ^**^ *p* < .01, ^***^ *p* < .001

## Table S11

*Standardized Results of RI-CLPMs with* Type=Complex: *Acculturation Preferences and Contact Out-of-School*

|  | Majority group | | | |  | Ethnic minority group | | | |
| --- | --- | --- | --- | --- | --- | --- | --- | --- | --- |
| WITHIN-PERSON Stability paths | T1→T2 | | T2→T3 | |  | T1→T2 | | T2→T3 | |
| Preference for maintenance (cen.) | .102 | | .097 | |  | .369 | | .383 | |
| Preference for maintenance (per.) | .028 | | .030 | |  | -.002 | | -.002 | |
| Preference for adoption (cen.) | .136 | | .002 | |  | .163 | | .182 | |
| Preference for adoption (per.) | .157 | | .151 | |  | -.016 | | -.017 | |
| Contact quantity | -.092 | | .054 | |  | .095 | | .097 | |
| Positive contact | .161 | | .133 | |  | -.121 | | -.069 | |
| Negative contact | .130 | | .126 | |  | -.068 | | -.100 | |
| WITHIN-PERSON Cross-lagged paths | T1→T2 | | T2→T3 | |  | T1→T2 | | T2→T3 | |
| Preference for maintenance (cen.) → Preference for maintenance (per.) | .066 | | .070 | |  | .238 | | .257 | |
| Preference for maintenance (cen.) → Preference for adoption (cen.) | -.160 | | -.156 | |  | .119 | | .128 | |
| Preference for maintenance (cen.) → Preference for adoption (per.) | -.043 | | -.042 | |  | .043 | | .034 | |
| Preference for maintenance (cen.) → Contact quantity | .094 | | .088 | |  | -.012 | | -.011 | |
| Preference for maintenance (cen.) → Positive contact | .005 | | .004 | |  | -.180 | | -.120 | |
| Preference for maintenance (cen.) → Negative contact | -.026 | | -.023 | |  | -.282 | | -.293 | |
|  |  | |  | |  |  | |  | |
| Preference for maintenance (per.) → Preference for maintenance (cen.) | .000 | | .000 | |  | .054 | | .054 | |
| Preference for maintenance (per.) → Preference for adoption (cen.) | .031 | | .030 | |  | -.062 | | -.065 | |
| Preference for maintenance (per.) → Preference for adoption (per.) | -.009 | | -.008 | |  | -.155 | | -.144 | |
| Preference for maintenance (per.) → Contact quantity | .011 | | .011 | |  | .112 | | .099 | |
| Preference for maintenance (per.) → Positive contact | .092 | | .080 | |  | .168 | | .109 | |
| Preference for maintenance (per.) → Negative contact | -.055 | | -.048 | |  | .142 | | .143 | |
|  |  | |  | |  |  | |  | |
| Preference for adoption (cen.) → Preference for maintenance (cen.) | -.119 | | -.110 | |  | -.044 | | -.047 | |
| Preference for adoption (cen.) → Preference for maintenance (per.) | .024 | | .023 | |  | -.027 | | -.030 | |
| Preference for adoption (cen.) → Preference for adoption (per.) | .024 | | .023 | |  | -.034 | | -.034 | |
| Preference for adoption (cen.) → Contact quantity | -.070 | | -.063 | |  | -.147 | | -.053 | |
| Preference for adoption (cen.) → Positive contact | -.107 | | -.091 | |  | -.142 | | -.098 | |
| Preference for adoption (cen.) → Negative contact | -.118^*^ | | -.100^*^ | |  | -.028 | | -.031 | |
|  |  | |  | |  |  | |  | |
| Preference for adoption (per.) → Preference for maintenance (cen.) | -.050 | | -.047 | |  | -.133 | | -.149 | |
| Preference for adoption (per.) → Preference for maintenance (per.) | -.029 | | -.031 | |  | -.215 | | -.320^**^ | |
| Preference for adoption (per.) → Preference for adoption (cen.) | .046 | | .045 | |  | -.249^*^ | | -.292^*^ | |
| Preference for adoption (per.) → Contact quantity | .032 | | .029 | |  | -.109 | | -.107 | |
| Preference for adoption (per.) → Positive contact | .005 | | .004 | |  | -.067 | | -.048 | |
| Preference for adoption (per.) → Negative contact | .036 | | .031 | |  | -.108 | | -.122 | |
|  |  | |  | |  |  | |  | |
| Contact quantity → Preference for maintenance (cen.) | .013 | | .012 | |  | .065 | | .076 | |
| Contact quantity → Preference for maintenance (per.) | .009 | | .010 | |  | .079 | | .096 | |
| Contact quantity → Preference for adoption (cen.) | -.083 | | -.083 | |  | -.086 | | -.104 | |
| Contact quantity → Preference for adoption (per.) | -.051 | | -.050 | |  | -.030 | | -.033 | |
| Contact quantity → Positive contact | .011 | | .010 | |  | -.176 | | -.133 | |
| Contact quantity → Negative contact | -.036 | | -.033 | |  | -.055 | | -.065 | |
|  |  | |  | |  |  | |  | |
| Positive contact → Preference for maintenance (cen.) | .048 | | .043 | |  | -.133 | | -.118 | |
| Positive contact → Preference for maintenance (per.) | .087 | | .087 | |  | .003 | | .003 | |
| Positive contact → Preference for adoption (cen.) | .057 | | .053 | |  | -.123 | | -.114 | |
| Positive contact → Preference for adoption (per.) | .065 | | .060 | |  | -.223 | | -.182 | |
| Positive contact → Contact quantity | .249^**^ | | -.061 | |  | -.215^*^ | | -.167^*^ | |
| Positive contact → Negative contact | -.003 | | -.002 | |  | .082 | | .072 | |
|  |  | |  | |  |  | |  | |
| Negative contact → Preference for maintenance (cen.) | -.014 | | -.015 | |  | -.107 | | -.157 | |
| Negative contact → Preference for maintenance (per.) | -.064 | | -.076 | |  | -.036 | | -.055 | |
| Negative contact → Preference for adoption (cen.) | -.048 | | -.052 | |  | -.161 | | -.246 | |
| Negative contact → Preference for adoption (per.) | .021 | | .023 | |  | -.231^*^ | | -.314^*^ | |
| Negative contact → Contact quantity | -.099 | | -.102 | |  | -.117 | | -.151 | |
| Negative contact → Positive contact | -.036 | | -.035 | |  | -.202 | | -.192 | |
| WITHIN-PERSON Correlations and Residual Covariances | T1 | T2 | | T3 |  | T1 | T2 | | T3 |
| Preference for maintenance (cen.) ↔ Preference for maintenance (per.) | .379^***^ | .388^***^ | | .459^***^ |  | .726^***^ | .721^***^ | | .823^***^ |
| Preference for maintenance (cen.) ↔ Preference for adoption (cen.) | -.203^*^ | -.154 | | -.164^*^ |  | -.050 | -.126 | | -.023 |
| Preference for maintenance (cen.) ↔ Preference for adoption (per.) | -.083 | -.010 | | -.011 |  | -.066 | .001 | | .001 |
| Preference for maintenance (cen.) ↔ Contact quantity | .173^**^ | .078 | | .078 |  | .271 | -.027 | | -.025 |
| Preference for maintenance (cen.) ↔ Positive contact | .169^*^ | .117^*^ | | .113^*^ |  | -.110 | -.123 | | -.082 |
| Preference for maintenance (cen.) ↔ Negative contact | -.019 | .009 | | .008 |  | -.205 | -.015 | | -.016 |
| Preference for maintenance (per.) ↔ Preference for adoption (cen.) | .018 | .076 | | .092 |  | -.107 | -.044 | | -.052 |
| Preference for maintenance (per.) ↔ Preference for adoption (per.) | .028 | .012 | | .014 |  | -.061 | -.096 | | -.099 |
| Preference for maintenance (per.) ↔ Contact quantity | .164^*^ | .098^*^ | | .111^*^ |  | .279^*^ | -.002 | | -.002 |
| Preference for maintenance (per.) ↔ Positive contact | .152^*^ | .134^**^ | | .145^**^ |  | .008 | -.135 | | -.095 |
| Preference for maintenance (per.) ↔ Negative contact | -.069 | -.027 | | -.030 |  | .000 | .022 | | .025 |
| Preference for adoption (cen.) ↔ Preference for adoption (per.) | .573^***^ | .434^***^ | | .474^***^ |  | .453^**^ | .489^***^ | | .490^***^ |
| Preference for adoption (cen.) ↔ Contact quantity | -.025 | -.011 | | -.011 |  | -.107 | -.190 | | -.180 |
| Preference for adoption (cen.) ↔ Positive contact | -.061 | .010 | | .010 |  | -.146 | .112 | | .076 |
| Preference for adoption (cen.) ↔ Negative contact | .079 | -.011 | | -.011 |  | -.003 | -.033 | | -.037 |
| Preference for adoption (per.) ↔ Contact quantity | -.038 | .020 | | .021 |  | -.004 | -.081 | | -.068 |
| Preference for adoption (per.) ↔ Positive contact | -.118 | -.024 | | -.023 |  | -.047 | .157 | | .095 |
| Preference for adoption (per.) ↔ Negative contact | .084 | .025 | | .024 |  | -.065 | -.113 | | -.109 |
| Contact quantity ↔ Positive contact | .561^***^ | .504^***^ | | .464^***^ |  | .380^*^ | .054 | | .392^**^ |
| Contact quantity ↔ Negative contact | -.078 | -.109 | | -.101 |  | -.092 | -.407^***^ | | -.376^***^ |
| Positive contact ↔ Negative contact | -.247^***^ | -.328^***^ | | -.293^***^ |  | -.061 | -.319^*^ | | -.492^***^ |
| BETWEEN-PERSON Correlations |  | | | |  |  | | | |
| Preference for maintenance (cen.) ↔ Preference for maintenance (per.) | .624^***^ | | | |  | .512 | | | |
| Preference for maintenance (cen.) ↔ Preference for adoption (cen.) | -.425^**^ | | | |  | .248 | | | |
| Preference for maintenance (cen.) ↔ Preference for adoption (per.) | -.153 | | | |  | .039 | | | |
| Preference for maintenance (cen.) ↔ Contact quantity | .019 | | | |  | -.598 | | | |
| Preference for maintenance (cen.) ↔ Positive contact | .303^**^ | | | |  | .017 | | | |
| Preference for maintenance (cen.) ↔ Negative contact | -.352^**^ | | | |  | .779 | | | |
| Preference for maintenance (per.) ↔ Preference for adoption (cen.) | -.011 | | | |  | .330 | | | |
| Preference for maintenance (per.) ↔ Preference for adoption (per.) | -.139 | | | |  | .232 | | | |
| Preference for maintenance (per.) ↔ Contact quantity | -.010 | | | |  | -.464 | | | |
| Preference for maintenance (per.) ↔ Positive contact | .069 | | | |  | -.018 | | | |
| Preference for maintenance (per.) ↔ Negative contact | -.133 | | | |  | .309 | | | |
| Preference for adoption (cen.) ↔ Preference for adoption (per.) | .832^***^ | | | |  | .871^***^ | | | |
| Preference for adoption (cen.) ↔ Contact quantity | -.072 | | | |  | .648^**^ | | | |
| Preference for adoption (cen.) ↔ Positive contact | -.289^**^ | | | |  | .583^***^ | | | |
| Preference for adoption (cen.) ↔ Negative contact | .493^***^ | | | |  | -.060 | | | |
| Preference for adoption (per.) ↔ Contact quantity | .085 | | | |  | .911^**^ | | | |
| Preference for adoption (per.) ↔ Positive contact | -.178 | | | |  | .728^***^ | | | |
| Preference for adoption (per.) ↔ Negative contact | .379^**^ | | | |  | -.178 | | | |
| Contact quantity ↔ Positive contact | .579^***^ | | | |  | .893^***^ | | | |
| Contact quantity ↔ Negative contact | .124 | | | |  | -.110 | | | |
| Positive contact ↔ Negative contact | -.576^***^ | | | |  | -.791^***^ | | | |

*Note:* cen. = Central domains; per. = Peripheral domains; *T* = time. Grey shaded values lost significance when accounting for the nested structure of the data, while blue-shaded values became significant in the clustered models.

^*^ *p* < .05; ^**^ *p* < .01, ^***^ *p* < .001

**References**

Byrne, B. M. (2012). *Structural equation modeling with Mplus: Basic concepts, applications, and programming.* Routledge.

Chen, N. F., Curran, P. J., Bollen, K. A., Kirby, J., & Paxton, P. (2008). An empirical evaluation of the use of fixed cutoff points in RMSEA test statistic in structural equation models. *Sociological Methods & Research*, *36*(4), 462–494. <https://doi.org/10.1177/0049124108314720>

Cheung, G. W., & Rensvold, R. B. (2002). Evaluating goodness-of-fit indexes for testing measurement invariance. Structural Equation Modeling, 9(2), 233–255. [https://doi.org/10.1207/S15328007SEM0902_5](https://psycnet.apa.org/doi/10.1207/S15328007SEM0902_5)

Cohen, J. (1988). Statistical power analysis for the behavioral sciences (2nd ed.). Lawrence Erlbaum Associates.

Little, T. D. (2013). *Longitudinal Structural Equation Modeling*. Guilford Press.

Satorra, A., & Bentler, P. M. (2001). A scaled difference chi-square test statistic for moment structure analysis. *Psychometrika, 66*(4), 507–514. <https://doi.org/10.1007/BF02296192>

Van de Schoot, R., Lugtig, P., & Hox, J. (2012). A checklist for testing measurement invariance. *European Journal of Developmental Psychology*, *9*(4), 486-492. https://doi.org/10.1080/17405629.2012.686740

1. Clustering at the school level was not possible due to the low number of schools (*n* = 7) involved in the project.
